# Heteroleptic metallosupramolecular aggregates*/*complexation for supramolecular catalysis

**DOI:** 10.3762/bjoc.18.62

**Published:** 2022-05-27

**Authors:** Prodip Howlader, Michael Schmittel

**Affiliations:** 1 Center of Micro- and Nanochemistry and (Bio)Technology, Universität Siegen, Organische Chemie I, Adolf-Reichwein-Str. 2, D-57068 Siegen, Germany

**Keywords:** heteroleptic complexation, information science, supramolecular catalysis, switching catalysis, systems chemistry

## Abstract

Supramolecular catalysis is reviewed with an eye on heteroleptic aggregates/complexation. Since most of the current metallosupramolecular catalytic systems are homoleptic in nature, the idea of breaking/reducing symmetry has ignited a vivid search for heteroleptic aggregates that are made up by different components. Their higher degree of functional diversity and structural heterogeneity allows, as demonstrated by Nature by the multicomponent ATP synthase motor, a more detailed and refined configuration of purposeful machinery. Furthermore, (metallo)supramolecular catalysis is shown to extend beyond the single "supramolecular unit" and to reach far into the field and concepts of systems chemistry and information science.

## Introduction

Supramolecular catalysis [1–3] for most chemists is associated with a catalytically active capsule providing either activating groups or surface/volume properties for catalytic activation [4–12]. In the present short review, limited to discrete heteroleptic metallo-supramolecular ensembles [13–14], we will show that, in addition to the above-mentioned way, there are other diverse possibilities to profit from supramolecular protocols in catalysis [9].

Metal–ligand-based 2D and 3D self-assembled architectures have been extensively studied over the past decades [15–21]. While in the early years, the focus has been on the exploration of structural features, more recent advancements have led to a multitude of extremely useful functional applications (molecular recognition, ion sensing, catalysis, etc.) [9,22]. Since most of these structures are homoleptic in nature, i.e., they are constructed from a single type of ligand [4–22], the idea of breaking/reducing symmetry has ignited a vivid search for heteroleptic aggregates that are made up by different components. Their higher degree of functional diversity and structural heterogeneity should allow, as amply demonstrated by Nature, for instance, by the multicomponent ATP synthase motor [23], a more detailed and refined configuration of purposeful machinery [24].

For the preparation of heteroleptic aggregates, one must differentiate between dynamic (rapidly exchanging) and kinetically inert heteroleptic metal–ligand interactions. While the inert heteroleptic metal–ligand motifs often center about iridium, ruthenium, rhodium etc. [25], the dynamic ones are constructed using copper(I), zinc(II), cadmium(II), iron(II), palladium(II), etc. as metal ions due to their more rapid ligand exchange rates [24–26].

The strategies to prepare inert vs dynamic heteroleptic aggregates are quite different. While in the former often a step-by-step attachment of the different ligands to the metal centers under kinetic control is dominating, the formation of dynamic aggregates relies on effective self-sorting protocols under thermodynamic control [24].

In its initial definition, self-sorting describes the capability to distinguish "self" from "non-self" in a mixture of constituents [27–30], i.e., the formation of well-defined homomeric aggregates [31] instead of a random combination of constituents in the product. This definition was later extended to heteromeric complexes by Issacs’ classification [32] of two main categories: (a) social self-sorting, which involves the assembly of different species, and (b) narcissistic self-sorting, which only involves aggregation of the same component. Over the past few years, a variety of social self-sorting protocols has led to a significant number of self-sorted cages/assemblies that have demonstrated their potential to act as functional materials [30].

Various self-sorting protocols leading to quantitative formation of heteroaggregates under thermodynamic control have recently proven their capacity. A prominent procedure developed by Sauvage on the basis of topological control [33] has found ample use in the preparation of rotaxane-based machines and devices [34]. A key element is a macrocyclic phenanthroline with an endotopic binding site as it precludes homoleptic complex formation. A further principle, introduced by Lehn, uses maximum site occupancy to afford heteroleptic aggregates [35]. While this principle is limited, the charge-separation approach by Stang is of much wider use [36]. Probably, most contributions in the literature, though, are based on using steric constraints in heteroleptic aggregation, since a variety of heteroleptic aggregation protocols have been developed by Schmittel [37] (for copper(I), zinc(II), cadmium(II), mercury(II) ions) and Yoshizawa/Fujita [38] (for palladium(II) ion) that involve pyridine-derived ligands. Highly innovative are the approaches for terpyridine-based complexes by Chan using complementary ligand binding [39], sometimes combined with conformational regulation [40], and of Newkome/Li [41] applying mainly geometric complementarity [42]. Clever utilized shape complementarity [21] for building heteroleptic palladium(II) cages whereas Crowley developed a procedure to kinetically metastable cages using naked Pd^2+^ [43–44].

**Figure 1:**
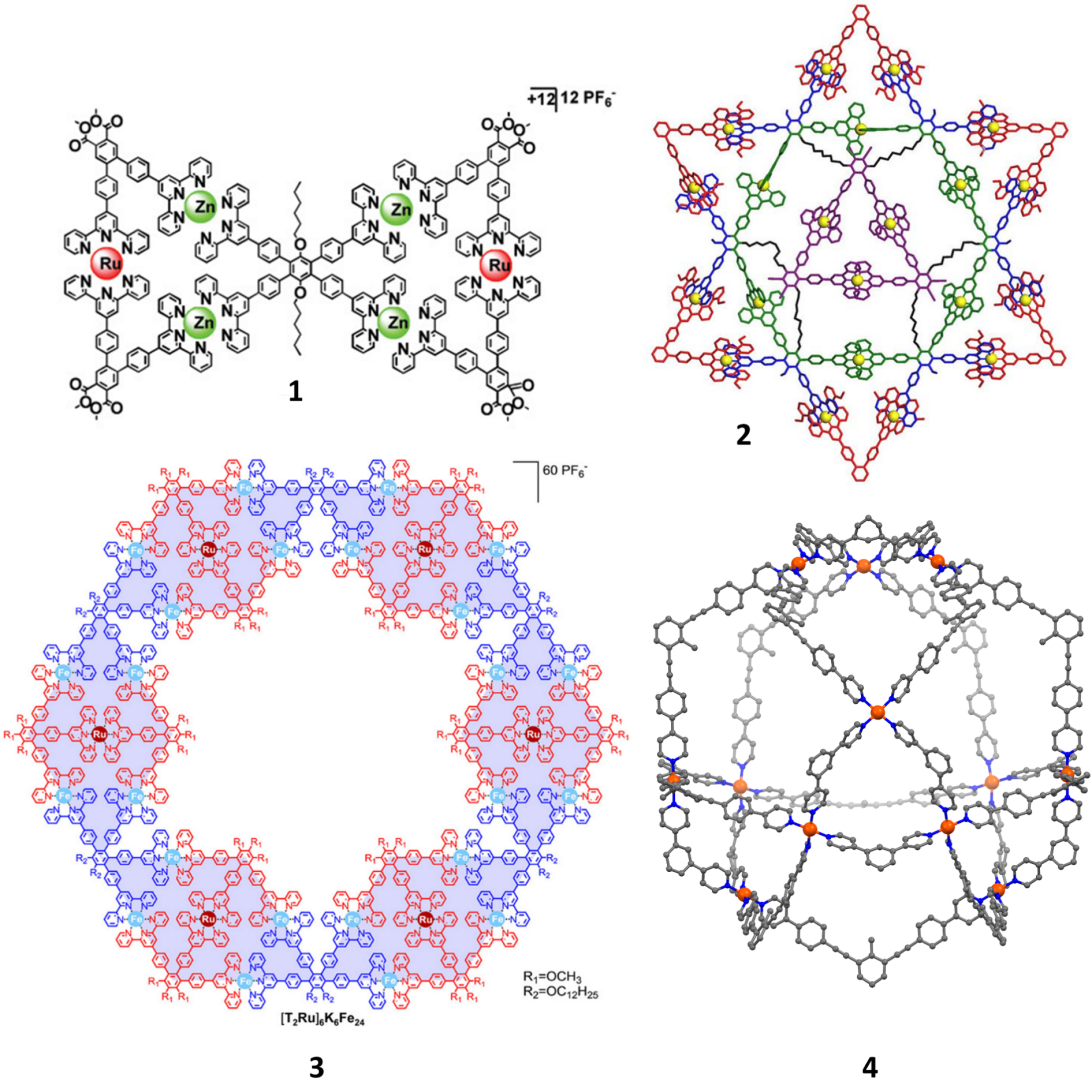
Butterfly **1** (Figure was reprinted with permission from [45]. Copyright 2012 American Chemical Society. This content is not subject to CC BY 4.0.). Six-pointed star **2** = [Cd_21_L_33_L’_6_] (Figure was reprinted with permission from [46]. Copyright 2019 American Chemical Society. This content is not subject to CC BY 4.0.). Hexagonal supramolecular nut **3** (Figure was reprinted with permission from [47]. Copyright 2016 American Chemical Society. This content is not subject to CC BY 4.0.). Cantellated tetrahedron **4** = Pd_12_L_12_L’_12_ (Fujita 2014 [48]).

The following selected structures (Figure 1) shall give a flavor of recent achievements in making fascinating heteroleptic structures using dynamic binding motifs.

The availability of powerful tools for building heteroleptic aggregates has led to a multitude of fascinating structures, accompanied by bright and confident prospects for interesting future applications. Concentrating on the topic of catalysis, however, one must confess that despite ample promises, so far, the number of established cases is rather small. For this brief account, we have identified four different categories under which the current examples describing supramolecular catalysis profiting from heteroleptic binding motifs may be summarized:

- Catalysis using heteroleptic discrete supramolecular architectures
- Catalytic effects due to nanomechanical motion
- Switchable catalysis due to reversible assembly/disassembly
- Toggling between intra- and intermolecular complexation in nanoswitches

## Review

### Catalysis using heteroleptic discrete supramolecular architectures

In its early years, supramolecular chemistry mainly focused on host–guest interactions, primarily on the electrostatic interaction of crown ethers and alkali metals [4]. While, in the beginning, crown ethers were an excellent choice for metal ion complexation, they later received ample recognition as supramolecular catalysts [49].

The majority of host capsules, however, has been constructed using aromatic walls that offer van-der-Waals and π–π stacking interactions in order to compensate for the absence of a strong electrostatic interaction. These non-covalent/ionic interactions play an important role in encapsulating aromatic organic molecules, especially in aqueous medium. Along this rationale, organic host molecules such as cyclodextrin [50], pillararenes, and cucurbiturils [51] have been developed in the last decades. Although, these exhibit excellent host–guest encapsulation properties with a variety of organic molecules, there are major drawbacks associated with them: variation of the shape and size of their cavity requires often tedious de-novo synthesis.

Eventually, these drawbacks have been tackled by synthesizing discrete supramolecular hosts based on metal–ligand coordination-driven self-assembly [15–22,52]. This approach not only solved the issue with low overall yields, but it also provided chemists with a superior control over the shape and size of the host’s cavity. Since the coordination bonds which define the host structure are labile in nature, they allow the formation of thermodynamically controlled architectures using a self-correcting mechanism [15,53]. Generally, the construction is spontaneous and highly selective with quantitative conversion.

In search of supramolecular cavity-induced catalysis, chemists have become fascinated toward the design of large and sophisticated molecular vessels. In this context, Stang [54], Nitschke [55], Fujita [56], and others [57] have reported several template-free assemblies giving access to novel structures. The primary objective of these nanovessels as supramolecular catalysts is to encapsulate organic reactant/s to lower the activation barrier, thereby mimicking the functions of enzymes (without replicating their structures). The structural dissimilarity between the reactants and the subsequent product often contributes to the successful release of the product from the reaction vessel, thus, reducing product inhibition. Hence, it can be envisioned that the introduction of functionality within the building blocks to decorate the inner cavity of the host would produce efficient types of catalyst, where the substrates get activated for a particular reaction upon interaction with the functional group inside the cavity.

**Figure 2:**
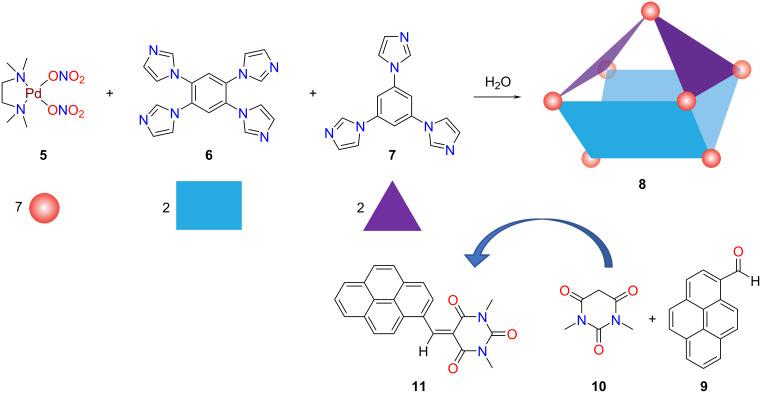
Synthesis of the three-component heteroleptic molecular boat **8** and its use as a catalyst for the Knoevenagel condensation reaction of **9** + **10**. Redrawn from [58].

Mukherjee et al. have demonstrated the construction of 3D nanocages employing imidazole-based multidentate donors [58]. The conformational asymmetry of the imidazole units opened the venue to nanocages of different shapes and sizes with ease. To take the directional self-assembly to the next level, a three-component self-assembly of the tetra- and tri-imidazole donors **6** and **7**, respectively, was carried out with **5** via social self-sorting to form the Pd_7_ molecular cage **8** (Figure 2) as driven by the directionality of the donor nitrogen of the building ligands. The unique three-component Pd_7_ molecular boat has a proper internal nanocavity showing preferential affinity towards aromatic molecules through π–π stacking with the hydrophobic aromatic wall of the host. Finally, the boat was investigated as a catalyst for the Knoevenagel condensation reaction (Figure 2) of a series of aromatic aldehydes with 1,3-dimethylbarbituric acid and Meldrum’s acid in aqueous media.

**Figure 3:**
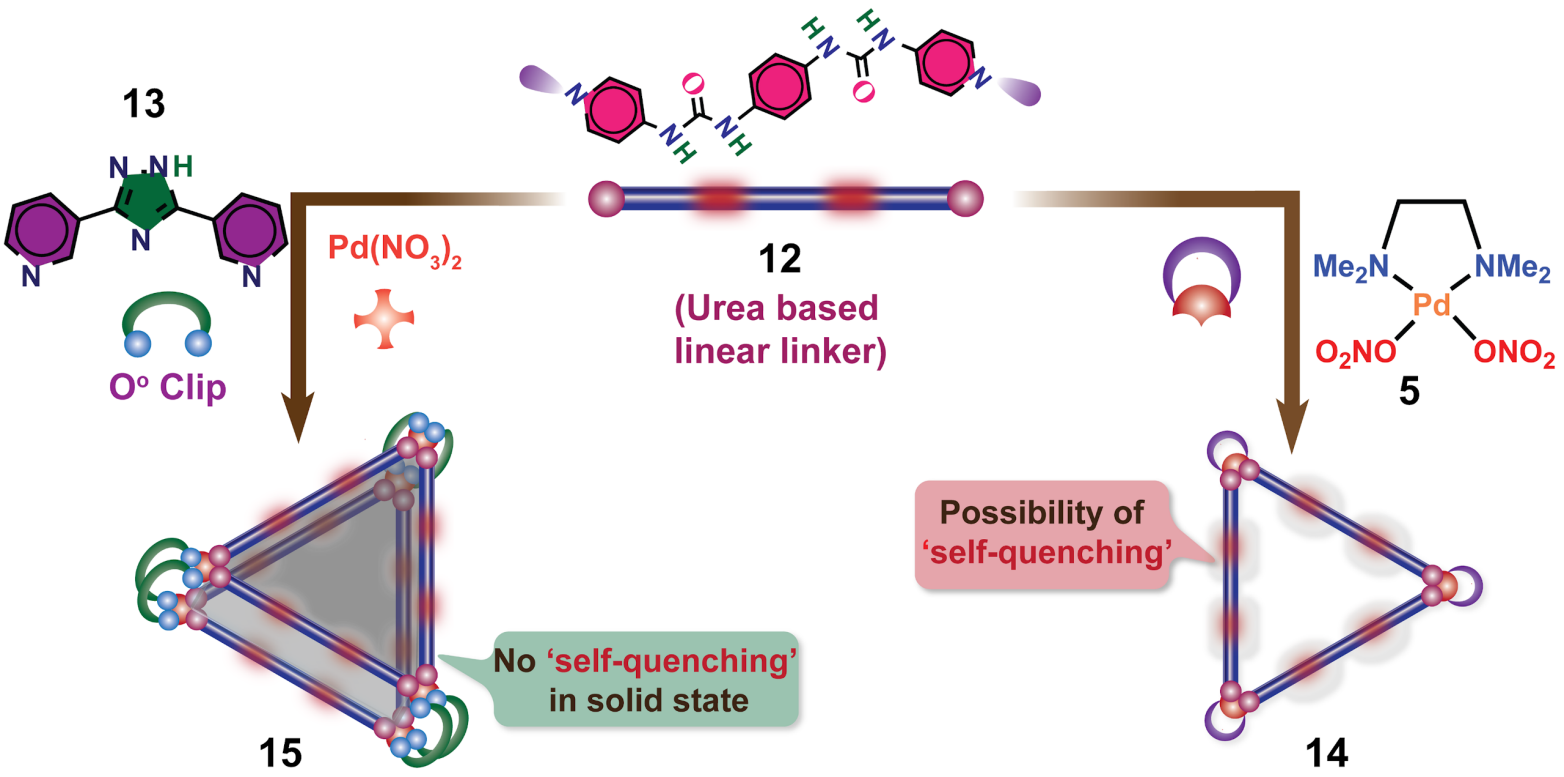
Synthesis of the two-component triangle **14** and three-component heteroleptic prism **15** [59]. Figure was adapted with permission from [59]. Copyright 2016 American Chemical Society. This content is not subject to CC BY 4.0.

One of the primary advantages of utilizing self-assembly to construct discrete nanocages with predetermined geometry and function is the use of a one-pot reaction employing complementary organic linkers with inorganic metal ions. Although the one-pot synthesis of homoleptic metallacages has been thoroughly investigated over the years [15–22] a more accurate understanding of the self-assembly of diverse components and the development of functionally integrated smart architectures for catalysis as “artificial enzymes” certainly demands further attention. Enticed by this idea, Mukherjee and co-worker have demonstrated the design and synthesis of urea-functionalized 2D/3D architectures and their catalytic activity. The urea moieties were incorporated within the building blocks and were meant to serve as binding sites for appropriate substrates, therefore, promoting selectivity and reactivity. For this purpose, they have chosen the ditopic bisurea “strut” **12** (Figure 3), which generated a 2D discrete molecular triangle **14** in the presence of an equimolar amount of the *cis*-(tmen)Pd(NO_3_)_2_ acceptor **5** [59].

**Figure 4:**
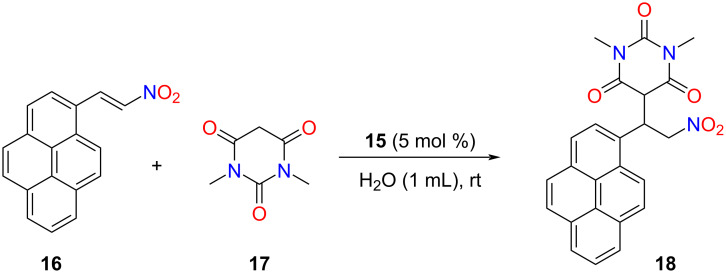
Catalytic Michael addition reaction using the urea-decorated molecular prism **15** [59].

In order to prevent the urea moieties on the triangle to get engaged in intermolecular H-bonding, which would lead to catalytic quenching, a unique design strategy was applied. Instead of using a *cis*-blocked palladium(II) unit for self-assembly, Pd(NO_3_)_2_ was employed along with the triazole-based 0° clip **13**. In a one-pot reaction, **12** and **13** in DMSO were treated with Pd(NO_3_)_2_ in a 1:1:1 ratio, which entailed the quantitative formation of the edge-directed molecular prism **15** (Figure 3). The adequate length of the ditopic clip **13** constrained the urea triangle from intersupramolecular H-bonding. Thus, the urea moieties in this newly assembled 3D architecture were freely available for interactions with appropriate guest molecules through H-bonding. Different organic molecules, such as nitroolefins, capable of forming H-bonding with the urea moieties inside the cavity were investigated in water for their ability to encapsulate in the cavity under heterogeneous conditions. Successful binding of the guest molecule was proven by UV–vis and IR spectroscopy. The multicomponent prism (Figure 3) was finally utilized as a heterogeneous catalyst for Michael and Diels–Alder (DA) reactions in water, representing an uncommon hydrogen-bond donating heterogeneous catalyst [59]. Intrigued by the successful guest-inclusion, Michael reactions were performed with prism **15** (Figure 4). Generally, most of the organocatalysts get destroyed during the work-up procedure, so that recovery is often diﬃcult. However, in the present case the catalyst could be easily recovered, and multiple catalytic cycles could be performed.

Supramolecular systems based on non-covalent interactions have drawn considerable attention in assembling efficient light harvesting systems (LHSs) in the last decade [60–61]. Significant attention has been centered to construct artificial LHSs via FRET (fluorescence resonance energy transfer) that include organic materials and supramolecular assemblies. Recently, FRET phenomena have been successfully demonstrated in supramolecular architectures based on metal organic frameworks and covalent organic frameworks [62–64]. However, poor solubility of such polymeric systems in common solvents restricts their use in potential applications. In this aspect, coordination-driven discrete architectures provide a promising future due to their facile one-pot synthesis and high solubility in common solvents.

**Figure 5:**
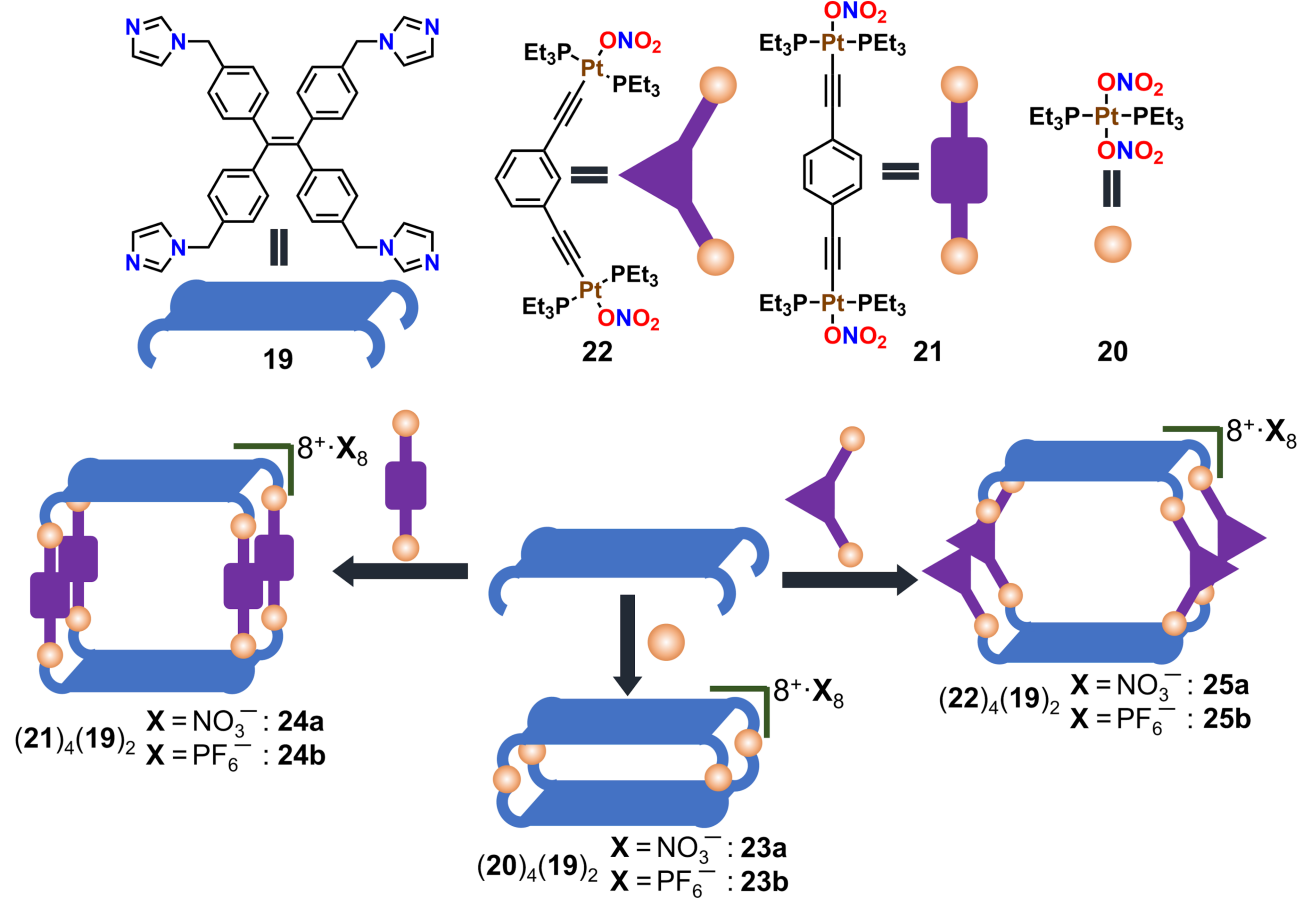
Self-assembly of two-component tetragonal prismatic architectures with different cavity size. Figure was adapted from [65]. (Published by the Royal Society of Chemistry, “Self-assembled metallasupramolecular cages towards light harvesting systems for oxidative cyclization“, © 2021 A. Kumar et al., distributed under the terms of the Creative Commons Attribution 3.0 Unported License, https://creativecommons.org/licenses/by/3.0).

**Figure 6:**
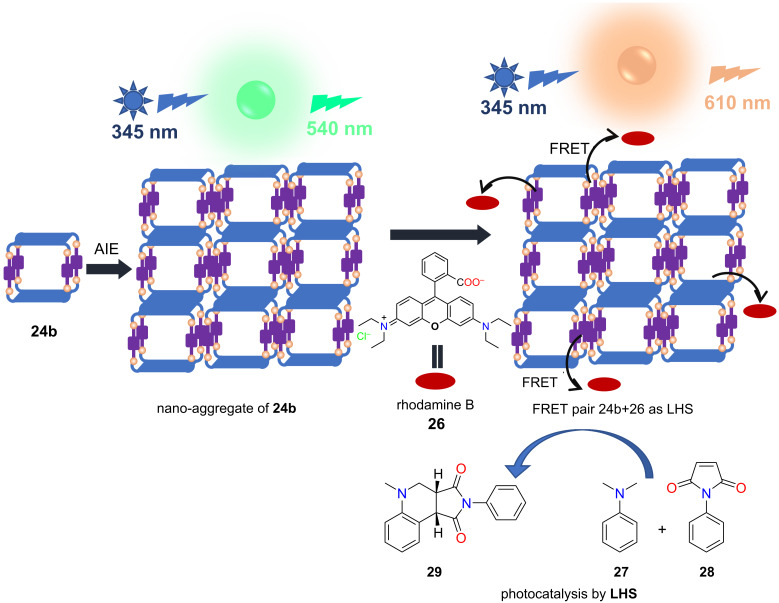
Construction of artificial LHS using rhodamine B as an acceptor and **24b** as donor generating a photocatalyst. Figure was adapted from [65]. (Published by the Royal Society of Chemistry, “Self-assembled metallasupramolecular cages towards light harvesting systems for oxidative cyclization“, © 2021 A. Kumar et al., distributed under the terms of the Creative Commons Attribution 3.0 Unported License, https://creativecommons.org/licenses/by/3.0).

Mukherjee and co-workers have developed supramolecular architectures containing tetraphenylethene (TPE) units which act as an aggregation-induced emissive (AIE) fluorophore [65]. The newly designed TPE-based tetraimidazole donor **19** has been treated with 180°/120° *trans*-Pt(II) acceptors which led to the coordination-driven self-assembly of 3D discrete molecular cages in aqueous medium (Figure 5). The 180° acceptor without an organic spacer, *trans*-[Pt(PEt_3_)_2_(ONO_2_)_2_] **20**, provided the cage **23a** with the molecular composition (**20**)_4_(**19**)_2_(NO_3_)_8_, whereas **21** (with a spacer unit) led to the formation of the Pt_8_ cage **24a** = (**21**)_4_(**19**)_2_(NO_3_)_8_. In a similar fashion the bent 120° acceptor **22** also assembled into the Pt_8_ cage **25a** = (**22**)_4_(**19**)_2_(NO_3_)_8_ [65]. Counter anion exchange from NO_3_^−^ to PF_6_^−^ made the cages soluble in acetonitrile. All three cages formed spherical supramolecular nano-aggregates in a water/acetonitrile (9:1) mixture and showed increased emission in the aggregated state [66]. Rhodamine B (**26**) was chosen as a FRET acceptor as there is a considerable overlap in energy of the donor emission and acceptor absorption. Then, artificial LHSs were constructed with aggregates from **24b** or **25b** at a donor/acceptor ratio of 5:1 (Figure 6). Finally, the light harvesting materials (**24b** + **26**) and (**25b** + **26**), respectively, were successfully employed as visible-light photocatalysts for a cross-coupling cyclization of *N*,*N*-dimethylaniline (**27**) and *N*-alkyl/aryl maleimides **28** (Figure 6). Notably, the systems showed much higher catalytic activity compared to similar reactions with the dye or cages alone.

Transition-metal catalysts play an important role for the development of intricate pharmaceutical drugs. Although transition-metal catalysts based on rhodium, cobalt, and palladium have been intensively studied, gold catalysis has received encouraging attention only recently [67–68]. Since selectivity of gold-catalyzed reactions is still a concern, the catalytic transformation is often controlled by introducing a ligand as a first coordination sphere of the active gold species. In a supramolecular approach, introduction of the ligated gold complex inside a hollow cage may significantly improve the reactivity and selectivity because the cage provides a second coordination sphere around the catalyst thus controlling the catalytic reaction. This idea has been impressively demonstrated by Reek [69], Ballester [70], and Raymond [71], where a single gold-based catalytic unit is encapsulated inside a molecular host to modulate the reactivity.

**Figure 7:**
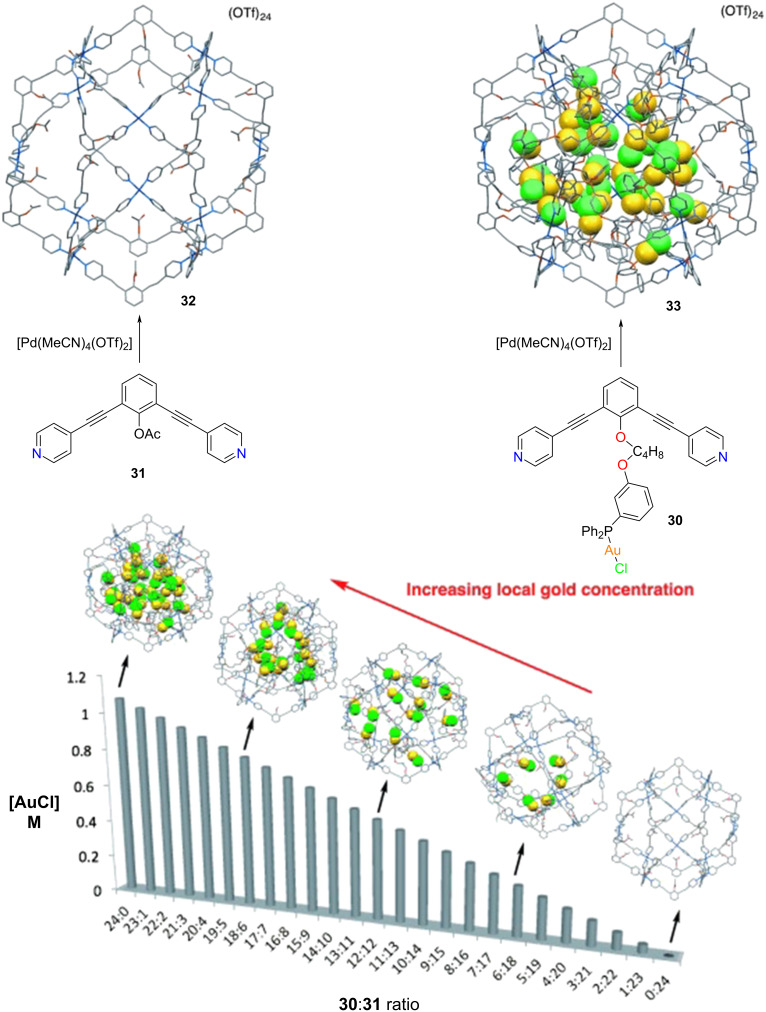
Synthesis of supramolecular spheres with varying [AuCl] concentration inside the cavity. Figure was adapted from [72], R. Gramage-Doria et al., “Gold(I) Catalysis at Extreme Concentrations Inside Self-Assembled Nanospheres”, Angewandte Chemie, International Edition, with permission from John Wiley and Sons. Copyright © 2014 Wiley-VCH Verlag GmbH & Co. KGaA, Weinheim. This content is not subject to CC BY 4.0.

**Figure 8:**
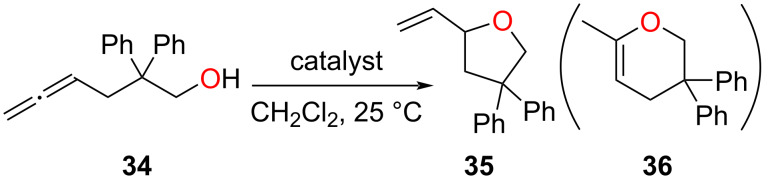
Hydroalkoxylation reaction of γ-allenol **34** in the presence of [AuCl]-encapsulated molecular spheres [72].

Recently, Reek and co-workers have constructed an M_12_L_24_ nanosphere by treating the bispyridyl 120° ligand **30** with a Pd(II) precursor [72]. Here, the ligand **30** is optimally functionalized with a phosphine gold(I) chloride moiety so that the metal catalyst will reside inside the sphere (Figure 7). In order to vary the local gold concentration inside the cavity, heteroleptic cages were assembled from a multicomponent one-pot reaction of Pd(II) with **30** and the analogous non-functionalized ligand **31**. By controlling the ratio of **30** and **31**, spheres with varying concentrations of AuCl could be constructed. Finally, the various spheres were investigated regarding the catalytic effect of the local gold concentration on the hydroalkoxylation of γ-allenol **34** (Figure 8). Since all catalytic tests were carried out at the same overall gold concentration of 5 mM, it was very interesting to observe that nanospheres with a very low local gold concentration could not catalyze the reaction at all. Actually, product formation was observed only with nanospheres having a higher **30**/**31** ratio (>6:18). Surprisingly, only the formation of the five-membered ring product **35** was observed, with a maximum yield of 88% at the highest **30**/**31** ratio of 24:0. The system was benchmarked with the building unit **30** itself and Ph_3_PAuCl where they observed a negligible conversion, which points toward the importance of local catalyst concentration.

**Figure 9:**
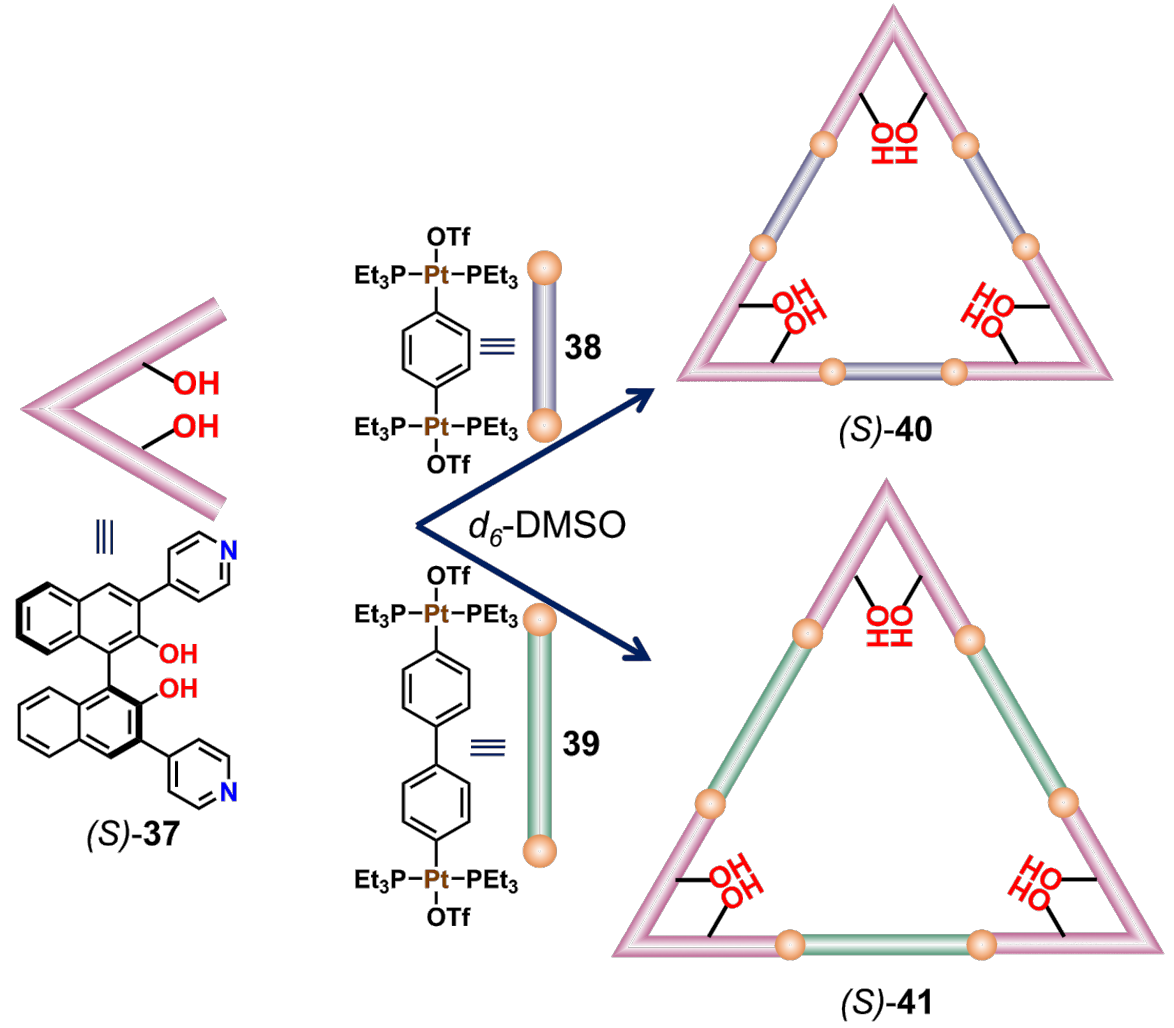
Two-component heteroleptic triangles of different size containing a BINOL functionality. Figure was adapted with permission from [75]. Copyright 2020 American Chemical Society. This content is not subject to CC BY 4.0.

A wide range of cavity-based artificial supramolecular catalysts has been successfully developed employing dynamic metal–ligand coordination bonds. While only a small fraction of these 3D architectures was useful for chiral catalysis, an even smaller fraction was able to provide a high stereoselectivity during asymmetric catalysis [5]. For instance, an enantiopure tetrahedral Pt_12_ cage has been previously studied for catalytic Michael addition reactions, but no enantioselectivity was detected because the chiral building blocks were located at peripheral positions thus not sufficiently breaking symmetry within the cavity [73]. Therefore, it was envisioned that the chiral moiety should be incorporated in the ligand unit in order to provide an enantiopure assembly with an asymmetric cavity. 1,1'-Binaphthol (BINOL) is one such chiral building block, which has been successfully utilized to carry out numerous asymmetric catalytic reactions [74]. Keeping this in mind, Stang and co-workers constructed heteroleptic triangles via the assembly of a BINOL-based ditopic ligand and 180° *trans*-Pt(II) acceptors. The 3,3'-dipyridyl-substituted chiral BINOL donor (*S*)-**37** has a bite angle of 60° and when treated with linear 180° acceptors **38** and **39**, it produced the differently sized triangles (*S*)-**40** and (*S*)-**41** depending on the length of the organic spacer in the acceptor unit (Figure 9) [75].

**Figure 10:**
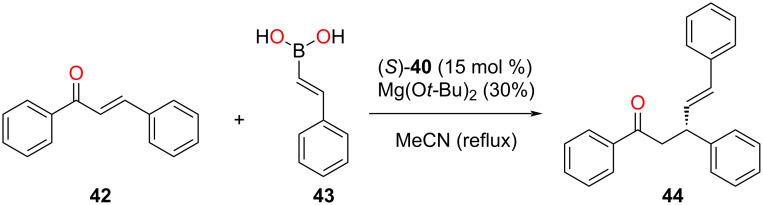
Asymmetric conjugate addition of chalcone **42** with *trans*-styrylboronic acid (**43**) catalyzed by BINOL-functionalized triangle (*S*)-**40** [75].

Since the interior cavity of the homochiral macrocycles was equipped with BINOL units, they were utilized as catalysts for the asymmetric conjugate addition of chalcone **42** with *trans*-styrylboronic acid (**43**, Figure 10). The catalytic reaction inside the chiral cavity of (*S*)-**40** provided a yield up to 91% with a very high enantioselectivity (94% ee). In contrast, the larger chiral macrocycle (*S*)-**41** afforded a slightly lower catalytic activity (87%), however, at a similar enantioselectivity (94% ee). Using similar reaction conditions, the non-assembled BINOL derivative (*S*)-3,3'-dibromo-[1,1'-binaphthyl]-2,2'-diol acted as a superior catalyst (99% yield) but achieved a lower enantioselectivity (84% ee). Therefore, this result indicates that the incorporation of multiple catalytic sites and an appropriate asymmetric cavity is the key for the enhancement of catalytic activity and stereoselectivity [75].

Strictly speaking, the above example does not contain fully dynamic heteroleptic metal fragments, as the aryl–Pt bond is not kinetically labile. Nevertheless, the example illustrates the opportunities in running enantioselective catalysis in mixed-ligand frameworks.

**Figure 11:**
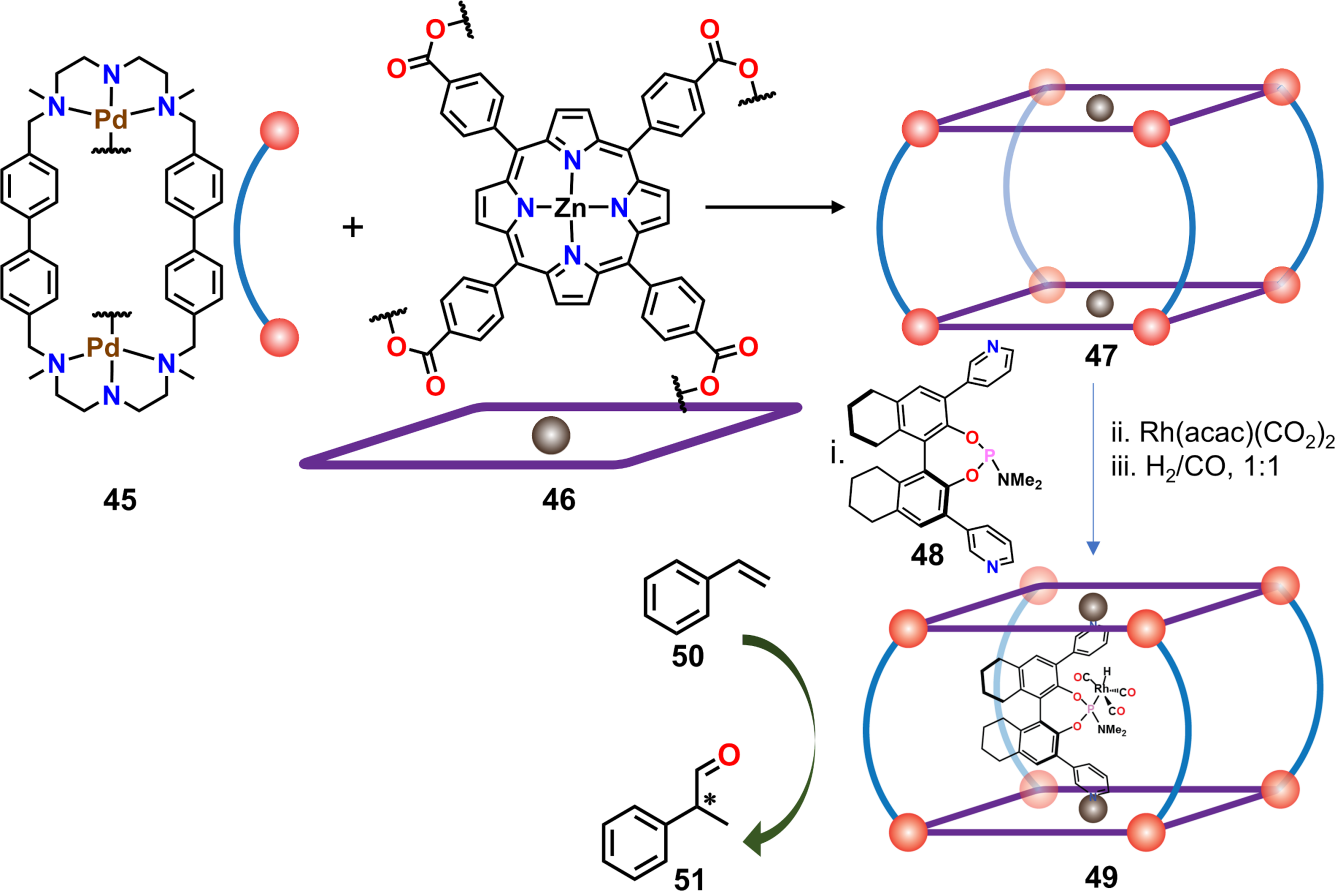
Encapsulation of monophosphoramidite-Rh(I) catalyst into a heteroleptic tetragonal prismatic cage **47** and its use in a stereoselective hydroformylation. Figure was adapted with permission from [77]. Copyright 2015 American Chemical Society. This content is not subject to CC BY 4.0.

Instead of constructing supramolecular catalysts by functionalization of the linker units, a different approach can be adopted where a catalytically active molecule is encapsulated inside a confined space, as demonstrated by Reek and co-workers by using a previously reported heteroleptic bisporphyrin cage [76]. The tetragonal prismatic nanocage **47** consisted of two zinc-porphyrin units along the two tetragonal faces (Figure 11), which allowed encapsulation of the chiral phosphoramidite **48** as a precursor for the final catalyst. In the next step, a transition-metal-ion based catalyst was prepared in situ by addition of one equiv of [Rh(acac)(CO)_2_], which generated the monoligated rhodium complex. Finally, catalysis was carried out at 5 bar of H_2_/CO (1:1) that converted the [Rh(acac)(CO)_2_] complex into the active hydride species **49** that is well known for hydroformylation reactions [77]. Incorporation of the monoligated catalyst into the confined cavity of the capsule showed very good catalytic activity towards the hydroformylation of styrene (**50**, Figure 11) with a high stereoselectivity (65% ee) at 32% conversion compared to the non-encapsulated catalyst, which only managed to yield 8% ee at 4% of conversion. Thus, the molecular capsule **47** can be viewed as a second coordination sphere of the catalyst, reminiscent of enzymatic active sites.

In summary, the above discrete molecular architectures containing cavity and sometimes smart functionality describe a new class of supramolecular catalysts that are effective tools to control the activity and selectivity of organic transformations. Clearly, building heteroleptic assemblies provides an unprecedented flexibility towards controlling the dimension as well as functionality. For instance, a particular catalytic reaction can be efficiently carried out by choosing a specific functionality. The functional entity responsible for catalysis can either be incorporated with the building blocks, or the catalyst itself may be encapsulated into the cavity. Unfortunately, the examples of heteroleptic cages acting as catalysts are limited so that further development is urgently needed.

### Catalytic effects due to nanomechanical motion

The above examples have demonstrated the potential of heteroleptic cages to enable catalysis under various conditions. In this subchapter, the role of the cage/architecture depends on its dynamics: the catalytic activity will correlate with the rate of thermal motion. As illustrated below, there are presently two phenomena known where continuous nanomechanical motion influences the catalytic activity: a) Increasing nanomechanical speed reduces product inhibition, and b) higher nanomechanical speed enlarges catalyst liberation.

**Figure 12:**
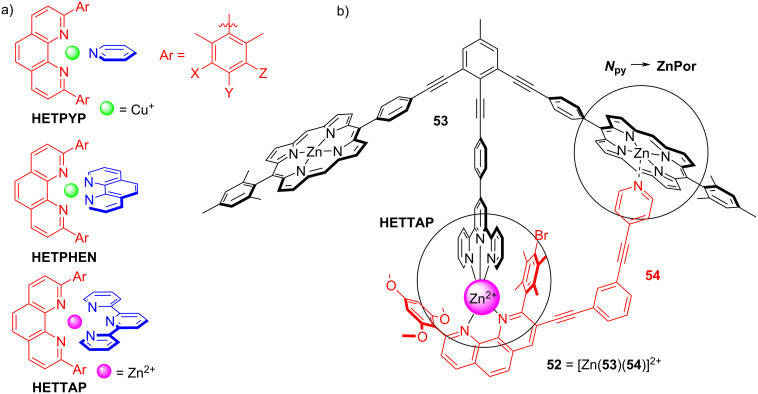
(a) Representations of the basic HETPYP, HETPHEN, and HETTAP complex motifs. (b) The three-component rotor **52** built on zinc(II) HETTAP and *N*_py_ → ZnPor coordination motifs [89].

While the development of multicomponent rotors has started almost 20 years ago with seminal works by Shionoya [78–80] and Kume [81–82], the fascinating prospects of discrete nanomechanical motion was impressively demonstrated by Aida with the development of multicomponent tweezers [83–84]. Using a variety of orthogonal complexation motifs, the Schmittel group has developed over the past five years a general approach to multicomponent rotors that relies on the binding difference found in the HETPYP (HETeroleptic PYridine and Phenanthroline complexes [85–86]), HETPHEN (HETeroleptic bisPHENanthroline complexes [87]) and HETTAP (HETeroleptic Terpyridine And Phenanthroline complexes [88]) interactions (Figure 12). Due to the different amount of donor atoms about the metal ion, the binding strength will decrease in the series of HETTAP > HETPHEN > HETPYP [37]. In any dynamic system designed for exchange motion predictably the weakest interaction will be the most dynamic one. This protocol is quite generally applicable and is readily illustrated by the ensuing example. The three-component rotor **52** = [Zn(**53**)(**54**)]^2+^ was developed on the basis of two complexation events, i.e., the zinc(II) HETTAP (log β = 14, log *K*_1_ ≥ 6) and *N*_py_ → ZnPor (log *K* = 4.45) binding motifs (Figure 12) [89]. Due to the design of **52**, the pyridine terminal oscillates between the two degenerate zinc porphyrin (ZnPor) stations of **53** at *k*_298_ = 24 kHz (at 298 K).

**Figure 13:**
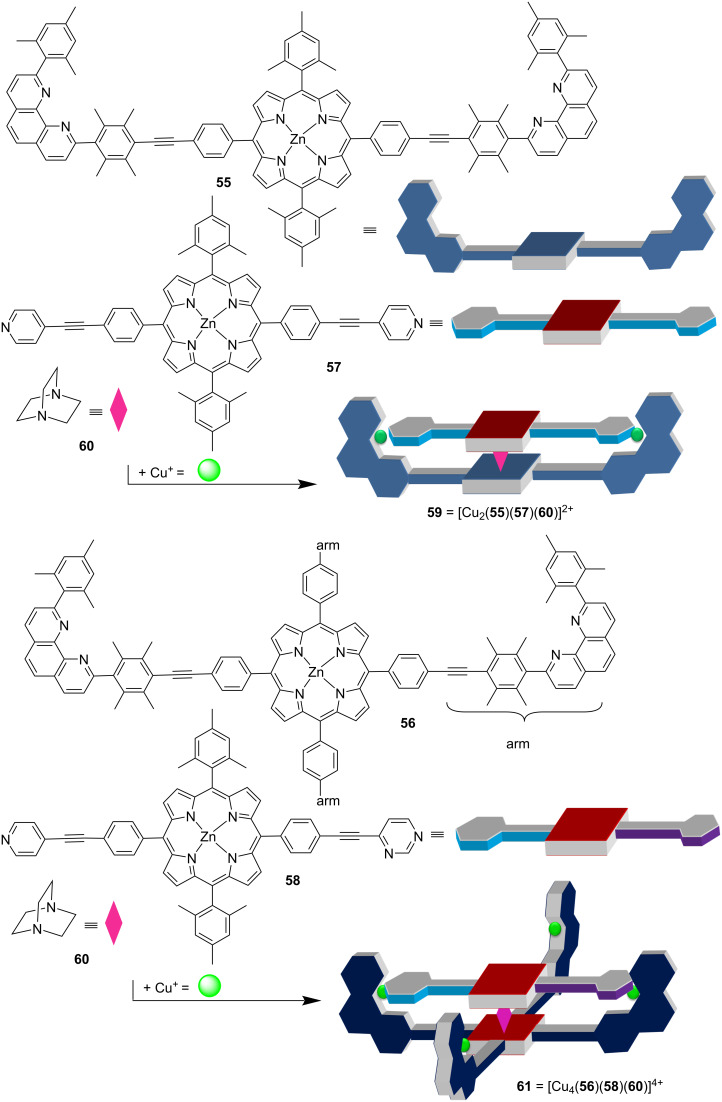
Two representative four-component rotors, with a (top) two-arm stator and (bottom) a four-arm stator. Figure was adapted with permission from [90]. Copyright 2013 American Chemical Society. This content is not subject to CC BY 4.0.

A similar exchange process was observed in the four-component rotors [90] developed along two orthogonal self-sorting motifs (HETPYP = *N*_py_ → [Cu(phenAr_2_)]^+^ and *N*_DABCO_ → ZnPor interactions). Again, the synthetic approach is a straightforward multicomponent self-sorting assembly. Accordingly, the distinct zinc porphyrins **55** and **57** were positioned at a defined distance through two *N*_DABCO_ → ZnPor interactions. Clearly, without additional measures, homo- and heteromeric assemblies would form. However, due to the additional HETPYP interaction(s) in the presence of copper(I) ions, thermodynamic stabilization quantitatively drives the reaction to the hetero-assembly **59**, simply by mixing the components in the correct stoichiometric ratio (Figure 13). Various nanorotor assemblies are possible by this approach.

The dynamics of the four-component rotor **59** = [Cu_2_(**55**)(**57**)(**60**)]^2+^ cannot be resolved due to the high symmetry in both the rotator and stator [91]. However, when rotator **58** with two different terminals was used then the rotational speed could be measured using VT ^1^H NMR [90]. Over the years, both the stator and the rotators were varied over a wide range, i.e., see **61**, changing the geometrical or constitutional situation at the binding sites [92]. In a detailed recent study, the finding of a Hammett correlation in such nanorotors corroborated that a rate-determining dissociation at the rotator–metal binding interaction dictated the rotational speed [93].

**Figure 14:**
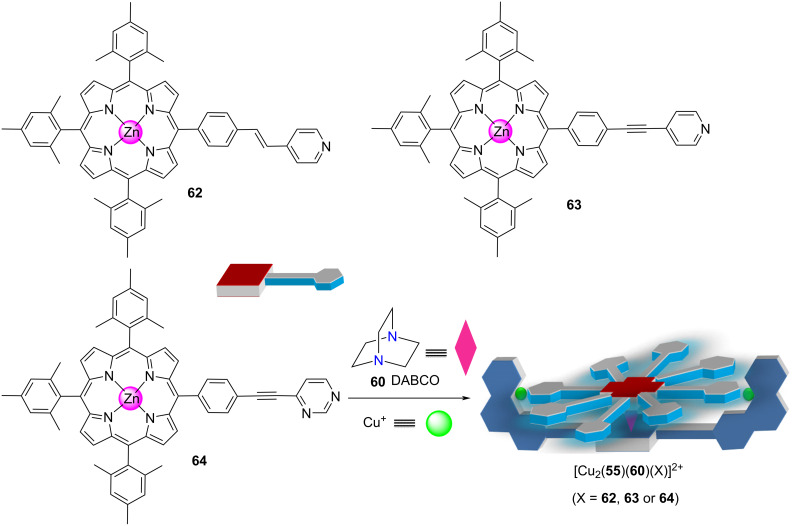
Four-component rotors with a monohead rotator. Figure was adapted with permission from [94]. Copyright 2018 American Chemical Society. This content is not subject to CC BY 4.0.

**Reducing Product Inhibition (RPI) through nanomechanical motion.** Recently, the suitability of the four-component rotors to act as catalysts in various click reactions was investigated having a look at nanorotors [Cu_2_(**55**)(**60**)(X)]^2+^ (with X = **62**, **63** or **64**), revealing an unexpected correlation between their rotational speed and catalytic activity [94] (Figure 14). Because in any moment of the rotation there should at least one copper(I) phenanthroline be freed from contact with the monodentate rotator X, one would expect that the temporarily exposed copper(I) ions are catalytically active. It is important to note that this copper ion due to steric impediments at the phenanthroline site will not engage in complexation with a second phenanthroline (see HETPYP concept [85–86]).

**Figure 15:**
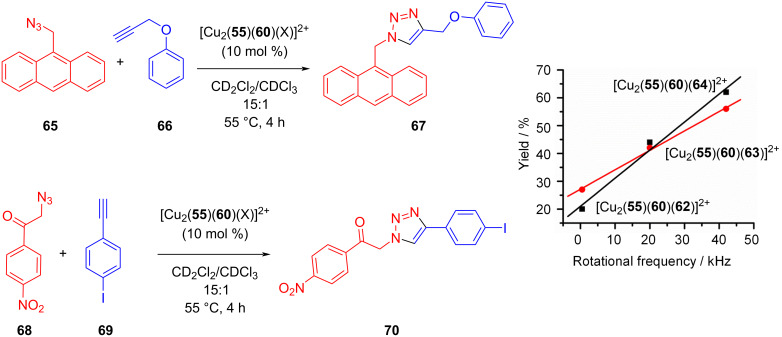
(left) Click reaction catalyzed by rotors [Cu_2_(**55**)(**60**)(X)]^2+^. (right) Yield as a function of the rotational frequency. Figure was adapted with permission from [94]. Copyright 2018 American Chemical Society. This content is not subject to CC BY 4.0.

The concept was probed by using nanorotors [Cu_2_(**55**)(**60**)(X)]^2+^ as catalyst (10 mol %) for the click reaction of 9-(azidomethyl)anthracene (**65**) and (prop-2-yn-1-yloxy)benzene (**66**) at 55 °C (4 h) [94]. Notably, the fastest nanorotor [Cu_2_(**55**)(**60**)(**64**)]^2+^ afforded the highest yield of the click product **67** (62%) followed by nanorotors [Cu_2_(**55**)(**60**)(**63**)]^2+^ (44%) and [Cu_2_(**55**)(**60**)(**62**)]^2+^ (20%) (Figure 15). The analogous tendency was recognized in a second click reaction, using now reactants **68** and **69** furnishing **70**. Markedly, the yield of both click reactions was linearly correlated with the exchange speed of the catalytic nanorotors (Figure 15, right). With faster rotational exchange of the nanorotor both the measured rate of catalysis at time = zero, *v*_0_, and the catalytic yield increased in a linear fashion. Such a clear correlation asks for a convincing theory. A straightforward explanation is that higher rotational speed should lead to a reduction of product inhibition by kicking out the product bound at the catalytic copper center. As shown experimentally, only the copper(I) phenanthroline unit which is temporarily not occupied by the rotator head is catalytically active. Thus, when the rotator dissociates from the copper(I) phenanthroline and moves to the product-filled site it should liberate the product into solution. The increased liberation of product with increasing speed of the nanorotor was proven by independent experiments, and additionally it was demonstrated that a “static” reference catalyst showed only a turnover of 1 due to product inhibition.

**Figure 16:**
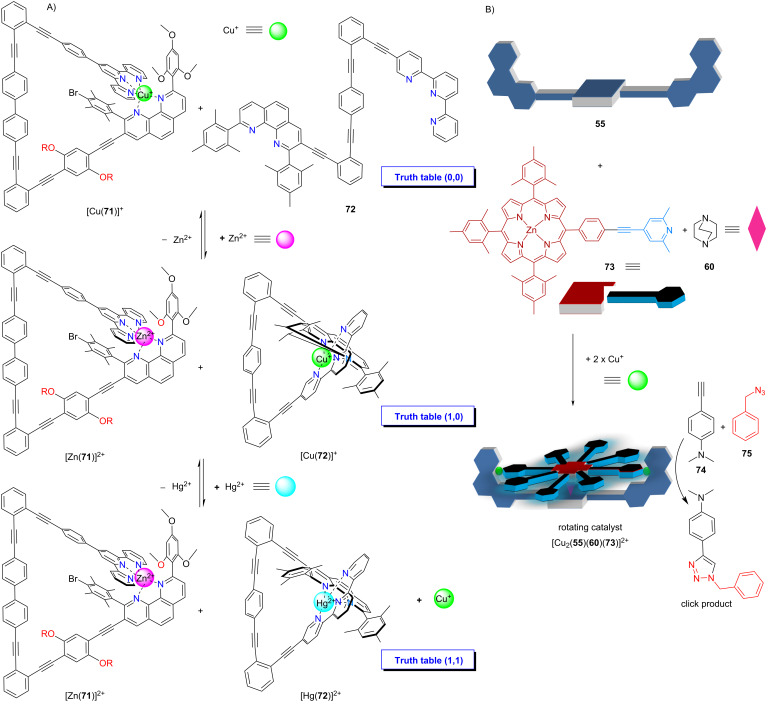
A supramolecular AND gate. a) In truth table state (0,0) two nanoswitches serve as the receptor ensemble. Inputs for the AND gate are Zn^2+^ and Hg^2+^. b) In truth table state (1,1), copper(I) ions are released that assemble the rotating catalyst [Cu_2_(**55**)(**60**)(**73**)]^2+^ the latter enabling the click reaction of **74** + **75**. For structure of compound **55**, see Figure 13. Figure was adapted with permission from [95]. Copyright 2020 American Chemical Society. This content is not subject to CC BY 4.0.

The capacity of the four-component nanorotors to act as catalyst in click reactions was more recently utilized for setting up a multicomponent logic AND gate that required the networking of altogether twelve components (Figure 16) [95]. At the heart of the logic operation, the ensemble of the copper(I)-loaded nanoswitch [Cu(**71**)]^+^ and ligand **72** was actuated by two metal-ion inputs (Zn^2+^ and Hg^2+^) and generated a stoichiometric Cu^+^ output according to the AND gate logic only in truth table state (1,1). The released copper(I) ions self-assembled the four-component rotor [Cu_2_(**55**)(**60**)(**73**)]^2+^ which enabled catalysis of a click reaction. In summary, copper(I) ions as stoichiometric output of the AND gate finally generated a catalytic output due to the assembly of a rotating four-component catalyst. Moreover, the AND gate could reversibly be reset into truth table state (0,0).

**Figure 17:**
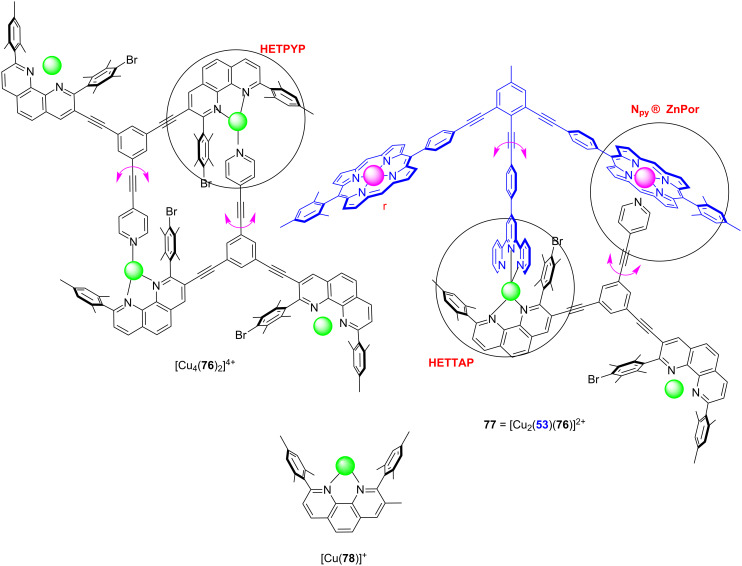
Two supramolecular double rotors (each has two rotational axes) and reference complex [Cu(**78**)]^+^ for catalysis. Figure is a derivative work from [96]. (Published by Wiley-VCH Verlag GmbH & Co. KGaA, Weinheim, © 2020 Goswami, A.; Schmittel, M. distributed under the terms of the Creative Commons Attribution 4.0 International License, https://creativecommons.org/licenses/by/4.0).

Like the nanorotors above, domino nanorotors with two exchanging rotational axes showed catalytic action that depended on the rotational speed [96]. Ligands **53** and **76** were conceived based on geometric complementarity at their coordination sites using the HETPYP interaction in both aggregates [Cu_4_(**76**)_2_]^4+^ and **77** = [Cu_2_(**53**)(**76**)]^2+^ (Figure 17). The structure of **76** suggested that the dimeric parallelogram-type double rotor [Cu_4_(**76**)_2_]^4+^ with two antiparallel pyridyl head groups operating as axles would form. Again, it is important to stress that the coordinatively frustrated terminal [Cu(phenAr_2_)]^+^ units will not engage in complexation with a second phenanthroline due to steric control in the HETPYP concept [85–86]. Since at a given time, only one of both HETPYP-bound pyridines in [Cu_4_(**76**)_2_]^4+^ can serve as axle, the rotor undergoes a domino rotation that was measured to occur at *k*_298_ = 142 kHz. For the heteromeric rotor [Cu_2_(**53**)(**76**)]^2+^, two orthogonal dynamic interactions are relevant, i.e., the weak *N*_py_ → ZnPor binding (log *K* = 4.3) and the stronger copper(I) HETTAP linkage (log β = 9.3). Ligand **53** with its terpyridine (tpy) and two ZnPor sites was designed in the way that the tpy should connect with **76** via a HETTAP binding motif while simultaneously allowing binding of the pyridine terminus of **76** to one of both ZnPor units of **53**. Now there are two motions possible that can only occur in a domino fashion. When the strong HETTAP complexation is intact, the exchange of the pyridine head of **76** between both ZnPor sites of **53** occurs at 64 kHz at rt. When the much stronger HETTAP complex dissociates, the much weaker *N*_py_ → ZnPor interaction remains intact and serves as a rotational axle, but now the exchange is slower by several orders of magnitude (*k*_298_ = 0.55 Hz).

The two rotors and reference complex [Cu(**78**)]^+^ (Figure 17) were compared for their catalytic activity in the click reaction of **79** + **80** (Table 1), after normalizing for their different content of copper [96]. Reactants **79** and **80** were chosen, because their click product **81** should be a rather good chelate ligand leading to product inhibition. The data given in Table 1 indicate that the rotor [Cu_2_(**53**)(**76**)]^2+^ has basically the same catalytic activity as a non-dynamic reference catalyst (turnover close to 1), because the exchange at the copper(I) sites preventing product inhibition is extremely slow (*k*_298_ = 0.55 Hz). The much faster rotation leading to an exchange of the *N*_py_ → ZnPor interaction is irrelevant for the catalysis. In contrast, the faster domino rotor [Cu_4_(**76**)_2_]^4+^ produced far superior yields (Table 1). The different yields correlate with the distinct *v*_0_ of the catalytic reaction. In conclusion, we see a clear trend in several rotating catalysts that with higher speed product inhibition is reduced (RPI).

**Table 1:** Yield of click product **81** using different catalysts [96]. Fast rotation leads to high yield.

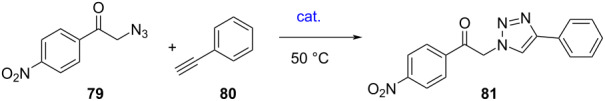			

catalyst	speed [Hz]	yield of **81**^a^	*v*_0_ [mol L^−1^ s^−1^]

[Cu_4_(**76**)_2_]^4+^	142 × 10^3^	63%	4.2 × 10^6^
[Cu_2_(**76**)(**77**)]^2+^	0.55	28%	1.8 × 10^6^
[Cu(**78**)]^+^ (static ref.)	0	26%	1.4 × 10^6^

^a^Yields determined from 3 independent runs.

**Figure 18:**
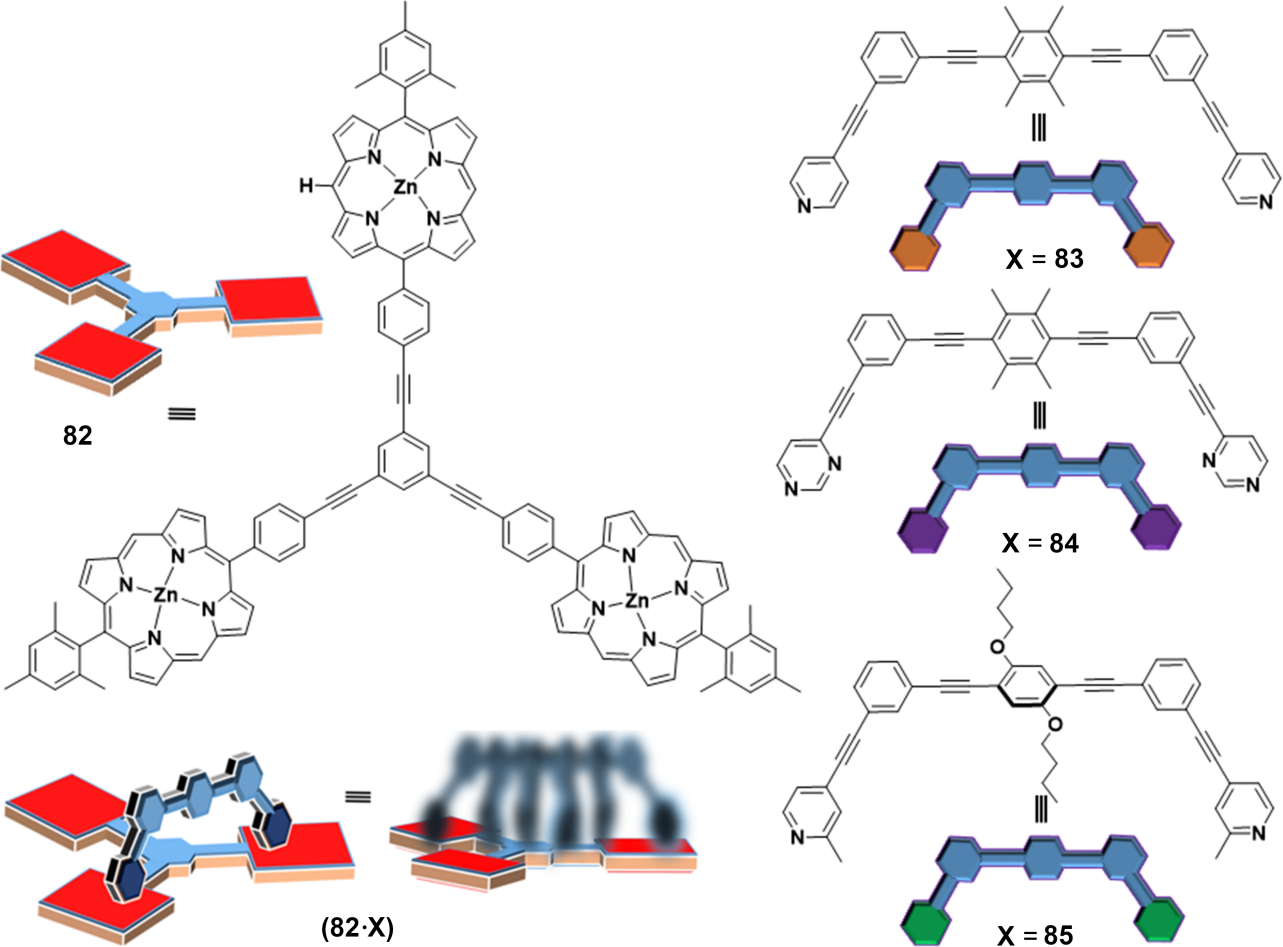
The slider-on-deck system (**82**•X) (X = **83**, **84**, or **85**). Figure is from [98] and was reprinted from the journal Angewandte Chemie, International Edition, with permission from John Wiley and Sons, (“Catalytic Three-Component Machinery: Control of Catalytic Activity by Machine Speed“ by Paul, I.; Goswami, A.; Mittal, N.; Schmittel, M.), Copyright © 2018 Wiley-VCH Verlag GmbH & Co. KGaA, Weinheim. This content is not subject to CC BY 4.0.

**Increased Liberation of Catalyst (ILC) through nanomechanical motion.** While the previous and related examples [97] are based on a reduction of product inhibition (RPI) with increasing speed of the rotating catalyst, there is a second general concept, i.e., ILC, for linking catalytic activity with the speed of a nanomechanical device. It is based on the increasing liberation of a bound organocatalyst with rising speed of the catalytic machinery. This concept was first realized in the slider-on-deck systems (**82**•X) (X = **83**, **84**, or **85**) (Figure 18) that were simply generated by mixing the tris-ZnPor deck **82** with one of the bipeds **83–85** (1:1) [98]. The thermal sliding speed of the biped across the deck **82** depends on the thermodynamic strength of the pyridine (or pyrimidine, methylpyridine) → ZnPor interactions of the biped’s feet with the ZnPor units. Obviously, weaker binding to ZnPor should lead to faster sliding [17].

**Figure 19:**
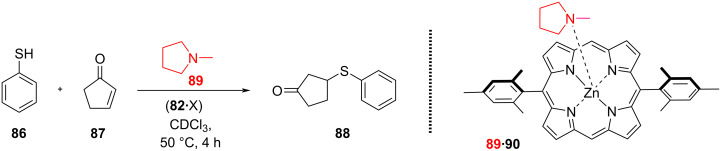
Catalysis of a conjugated addition reaction in the presence of the slider-on-deck system (**82**•X) (X = **83**, **84**, or **85**) [98]. No catalysis was observed with the static reference **89**•**90**.

With one of the three ZnPor units being available for the attachment (immobilization) of an organocatalyst, we wondered about the catalytic activity of the dynamic three-component ensembles **89**•(**82**•X) using *N*-methylpyrrolidine (**89**) as organocatalyst. For assessment, the conjugate addition of **86** and **87** was studied at different temperatures for 4 h (Figure 19). In comparison with the static reference system **89**•**90** (no yield at 50 °C), there was a clear trend for higher yields (Table 2) the faster the exchange process in the slider-on-deck is: system **89**•(**82**•**83**) furnished (18 ± 2)%, **89**•(**82**•**84**) afforded (32 ± 2)%, and **89**•(**82**•**85**) provided (50 ± 2%) of **88** at 50 °C after 4 h. Control experiments uncovered that product formation was kinetically controlled and that the slider-on-deck systems on their own were catalytically silent. With various controls one could demonstrate that the effect on catalysis in the catalytic machinery was due to kinetic and not thermodynamic reasons (Figure 19).

**Table 2:** Activation data (sliding motion) of the slider-on-deck systems (**82**•X) (X = **83**, **84**, or **85**) [98] and their performance in catalyzing the formation of **88** in presence of catalyst **89** (see Figure 19).

**Slider-on-deck**	(**82**•**83**)	(**82**•**84**)	(**82**•**85**)

∆*H^‡^* [kJ mol^–1^]	54.7 ± 0.5	45.5 ± 0.6	42.9 ± 0.6
∆*S^‡^* [J mol^–1^ K^–1^]	24.8 ± 2.2	10.1 ± 2.5	7.5 ± 2.5
∆*G^‡^*_298_ [kJ mol^–1^]	47.3	42.5	40.7
*k*_298_ [s^–1^]	32.2 × 10^3^	220 × 10^3^	440 × 10^3^
yield of **88** [%]	18 ± 2	32 ± 2	50 ± 2
liberated cat. **89**	29%	48%	76%

**Figure 20:**
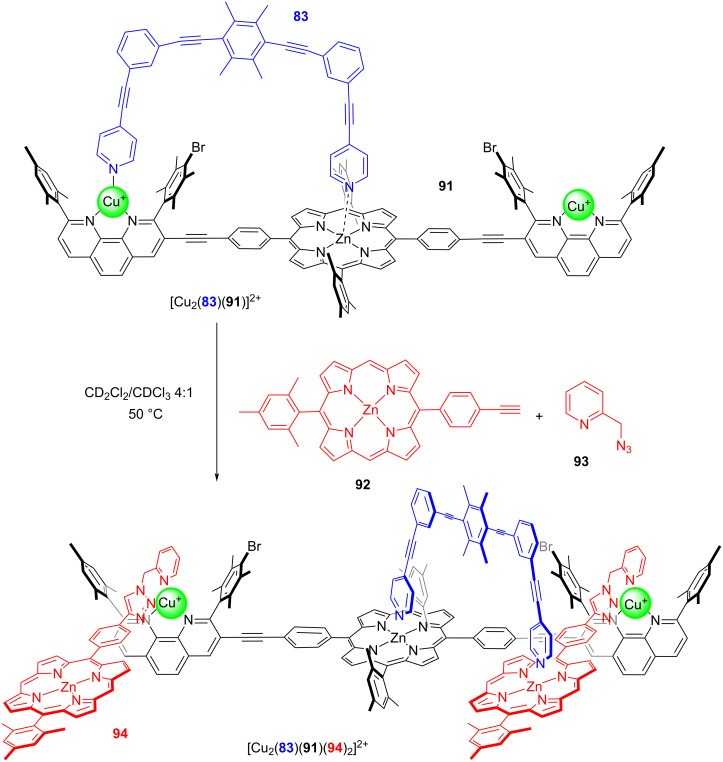
A rotating catalyst builds a catalytic machinery. For catalysis of the catalytic machinery, see Figure 21. Figure was adapted from [100] (“Evolution of catalytic machinery: three-component nanorotor catalyzes formation of four-component catalytic machinery“ by Goswami, A. et al., © The Royal Society of Chemistry 2021, distributed under the terms of the Creative Commons Attribution-NonCommercial 3.0 Unported License, https://creativecommons.org/licenses/by-nc/3.0/). This content is not subject to CC BY 4.0.

While the concept of increased liberation of an organocatalyst (ILC) has been demonstrated in other dynamic nanomechanical systems as well [99], a particular highlight was recently realized with a catalytic nanorotor that was able to build a new catalytic machinery [100]. Hereunto, the two concepts RPI and ILC were combined in a synergistic manner, starting with rotor [Cu_2_(**83**)(**91**)]^2+^ (*k*_298_ = 46.0 kHz) that catalyzed the click reaction between the zinc porphyrin ligand **92** and the azide **93** furnishing triazole **94** (Figure 20). As it was possible to drive this reaction to completion and as the click product **94** proved to be a good chelate ligand for the two copper(I) phenanthroline sites of [Cu_2_(**83**)(**91**)]^2+^, the formation of the dynamic four-component slider-on-deck [Cu_2_(**83**)(**91**)(**94**)_2_]^2+^ was warranted. The exchange of the biped **83** across the newly generated deck [Cu_2_(**83**)(**91**)(**94**)_2_]^2+^ occurred at a rate of *k*_298_ = 65.0 kHz.

**Figure 21:**
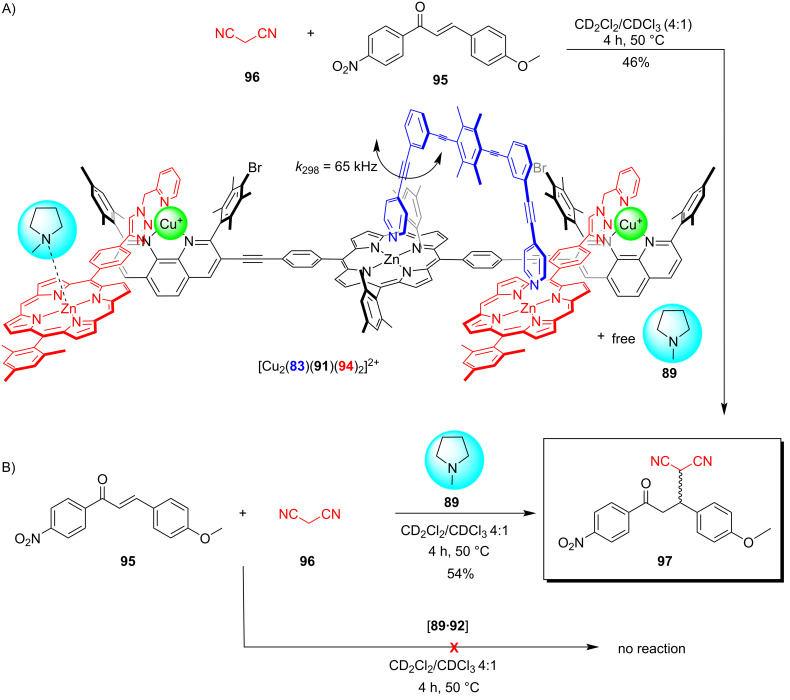
Catalytic machinery. Figure was adapted from [100] (“Evolution of catalytic machinery: three-component nanorotor catalyzes formation of four-component catalytic machinery“ by Goswami, A. et al., © The Royal Society of Chemistry 2021, distributed under the terms of the Creative Commons Attribution-NonCommercial 3.0 Unported License, https://creativecommons.org/licenses/by-nc/3.0/). This content is not subject to CC BY 4.0.

As known from earlier ILC work (vide supra ref. [98]), a carefully chosen organocatalyst can be immobilized at a ZnPor unit in a way that no catalysis will result (at a specific temperature and in a defined time). We thus started with the rotor [Cu_2_(**83**)(**91**)]^2+^ in the presence of ligand **92** as well as organocatalyst *N-*methylpyrrolidine (**89**) and the substrates **95** and **96** for a Michael addition (Figure 21). No formation of product **97** was observed, because the complex between the catalyst **89** and the zinc porphyrin **92** is catalytically inactive.

However, upon addition of azide **93**, the formation of [Cu_2_(**83**)(**91**)(**94**)_2_]^2+^ was detected. Since the organocatalyst originally firmly bound at the ZnPor of ligand **92** is now part of the dynamic slider-on-deck [Cu_2_(**83**)(**91**)(**94**)_2_]^2+^, the motion of biped **83** will dynamically release **89** into solution. Indeed, now the catalysis of addition product **97** was turned ON. While supramolecular transformations are widely recognized [101–103], the present example illustrates how a supramolecular catalyst (three-component rotor) transforms itself into a new catalytic machinery and turns on the respective catalytic process. All in all, this protocol is remotely reminiscent of gradual evolutionary processes [100].

In summary, both the RPI and ILC concepts using variable speed of nanomechanical machinery for catalytic effects are novel and innovative not only for supramolecular, but certainly also for common catalysis. In particular, product inhibition in catalysis is a frequently encountered challenge during catalyst development. Moreover, the RPI concept may point the way towards the development of endergonic catalytic transformations, because in many of those the product needs to be stabilized within the catalytic cavity. Release from the active site then requires destruction of the stabilizing interactions. For instance, Nature has chosen in the ATP-synthase to use “fueled” nanomechanical motion to release ATP from the active site [23].

### Switchable catalysis due to reversible assembly/disassembly

The common modus operandi to set up switchable catalysis usually relies on systems that can be toggled between two (or more) distinct switching states within a molecule [104–107]. In contrast, supramolecular approaches allow the shuffling and reshuffling of components to switch ON/OFF catalytic processes, a topic that has not yet found adequate attention, but links supramolecular catalysis to systems chemistry [108–109].

**Figure 22:**
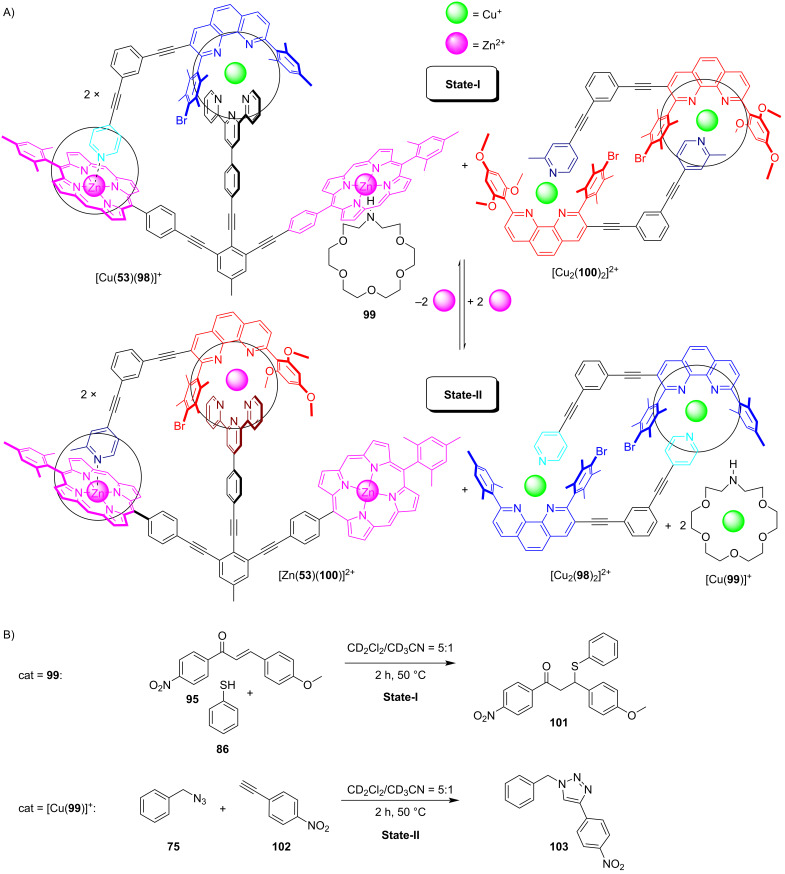
An information system based on (re)shuffling components between supramolecular structures [99]. Figure was adapted with permission from [99]. Copyright 2019 American Chemical Society. This content is not subject to CC BY 4.0.

The following information system utilizes a seven-component mixture that is reversibly reconfigured through fully reversible assembly and disassembly thereby tuning ON/OFF two diverse catalytic reactions [99]. Addition and removal of zinc(II) ions triggered altogether three diverse processes: i) mutual re-shuffling of components leading to two different nanorotors, ii) catalysis depending on decisively different exchange rates in the nanorotors, and iii) two different catalytic processes (Figure 22).

The key challenge in the toggling process was to interconvert two nanorotors by exchange of two components but with a single-input trigger from outside. Eventually, the two-component reshuffling was solved by providing zinc as trigger (component 1) and by using a ligand (component 2) from a ligand reservoir. In the initial self-sorted State-I, the rotor [Cu(**53**)(**98**)]^+^ was paired with [Cu_2_(**100**)_2_]^2+^, the latter representing a reservoir for the rotator arm **100**. Addition of zinc(II) ions induced a second self-sorting that encompassed transfer of two components along the following equation: 2 × [Cu(**53**)(**98**)]^+^ + [Cu_2_(**100**)_2_]^2+^ + 2 × Zn^2+^ → 2 × [Zn(**53**)(**100**)]^2+^ + [Cu_2_(**98**)_2_]^2+^ + 2 × Cu^+^. Accordingly, the added zinc(II) ions and ligand **100** from the reservoir [Cu_2_(**100**)_2_]^2+^ enabled the formation of the zinc(II)-based rotor [Zn(**53**)(**100**)]^2+^ (Figure 22a). The liberated ligand **98** reacted with the copper(I) ions to afford [Cu_2_(**98**)_2_]^2+^ representing again a reservoir for a rotator. Based on the stoichiometry of the above equation, two equiv of copper(I) ions were liberated. Since the released free copper(I) ions generated problems with reversibility of the transformation, 1-aza-18-crown-6 (**99**) (2.0 equiv) had to be added as a receptor for Cu^+^.

As State-I contains the azacrown ether **99**, a potential organocatalyst, and State-II harbors the copper complex [Cu(**99**)]^+^ a likely click catalyst, both networked states were expected to be catalytically active, possibly even in an ON/OFF manner [99]. To test for dual catalysis (Figure 22b), State-I was reacted at 50 °C with 1.0 equiv of catalyst **99** (with respect to rotor) and 10.0 equiv (with respect to rotor) of substrates **75**, **86**, **95**, and **102** in CD_2_Cl_2_/CD_3_CN 5:1 for 2 h. Analysis demonstrated that 30% of product **101** but no click product **103** had formed. State-II was furnished by addition of 1.0 equiv of zinc(II) ions (with respect to the rotor) and heated under identical conditions. Finally, 55% of the click product **103** was revealed, but without further conversion of product **101**. A notable reproducibility of the yields was identified in two consecutive catalytic cycles. As a result, an astounding switchable catalytic system could be based on information processing (Figure 22).

**Figure 23:**
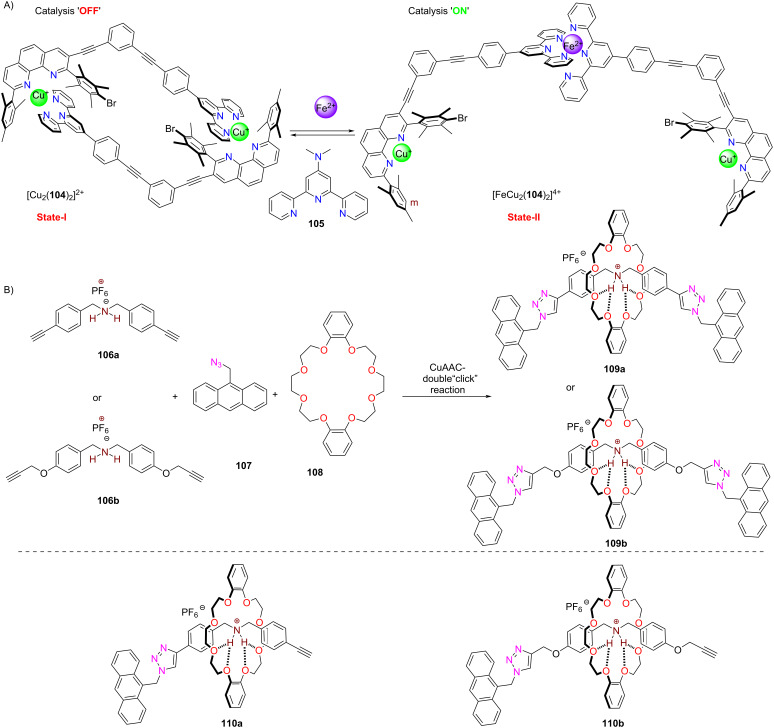
Switching between dimeric heteroleptic and homoleptic complex for OFF/ON catalytic formation of rotaxanes. Figure is a derivative work from [110]. (Published by Wiley-VCH Verlag GmbH, © 2021 Ghosh, A. et al. distributed under the terms of the CC By-NC 4.0 International License, https://creativecommons.org/licenses/by-nc/4.0). This content is not subject to CC BY 4.0.

The following example of switchable catalysis involves the interconversion of the closed dimeric parallelogram [Cu_2_(**104**)_2_]^2+^ and the bishomoleptic complex [FeCu_2_(**104**)_2_]^4+^ (Figure 23), the latter controlling a double-click catalytic access to rotaxanes **109**, by addition/removal of iron(II) ions [110]. Although [FeCu_2_(**104**)_2_]^4+^ is an open and flexible structure, the availability of two catalytic copper(I) centers positioned at 34 Å in the transition state of the second click reaction, leads to an astounding synthetic efficiency, although there is a major distance mismatch between the copper(I) ions in [FeCu_2_(**104**)_2_]^4+^ and two triazole units of the rotaxane.

For instance, the system [FeCu_2_(**104**)_2_]^4+^ (*d*_Cu–Cu_ = 34 Å) afforded the formation of both **109a** (73%) and **109b** (82%) in high yield (Figure 23b) [110], although the relevant distances in the pseudo-rotaxane **110a**,**b** (prior to the second click reaction) are 14.3 and 21.1 Å. Actually, the yield with [FeCu_2_(**104**)_2_]^4+^ was far better than with a dicopper reference catalyst, where the separation of the copper(I) ions was optimal (*d*_Cu–Cu_ = 14.6 Å) for the formation of **109a**. The findings were explained based on a model in which the monotriazoles **110a**,**b** were bound to both copper centers prior to the second click reaction. Aside of one copper(I)–triazole interaction, the model suggested a chelate cooperative effect resulting from an additional η^2^-binding of the second copper(I) at the acetylene unit at which the second click reaction would take place. In case of distance mismatch, it is the formation of this copper–alkyne η^2^-complex that compensates for the build-up of strain as demonstrated by DFT computations [110]. Once the second click reaction has occurred, the rotaxane is liberated under release of strain in the catalyst if there was a distance mismatch. For cases with a close match of distances, product inhibition reduced the yield. Due to the high relevance of the CuAAC approach [111] for the preparation of rotaxanes, the exploitation of cooperative and strain effects in double-click strategies is a promising strategy.

**Figure 24:**
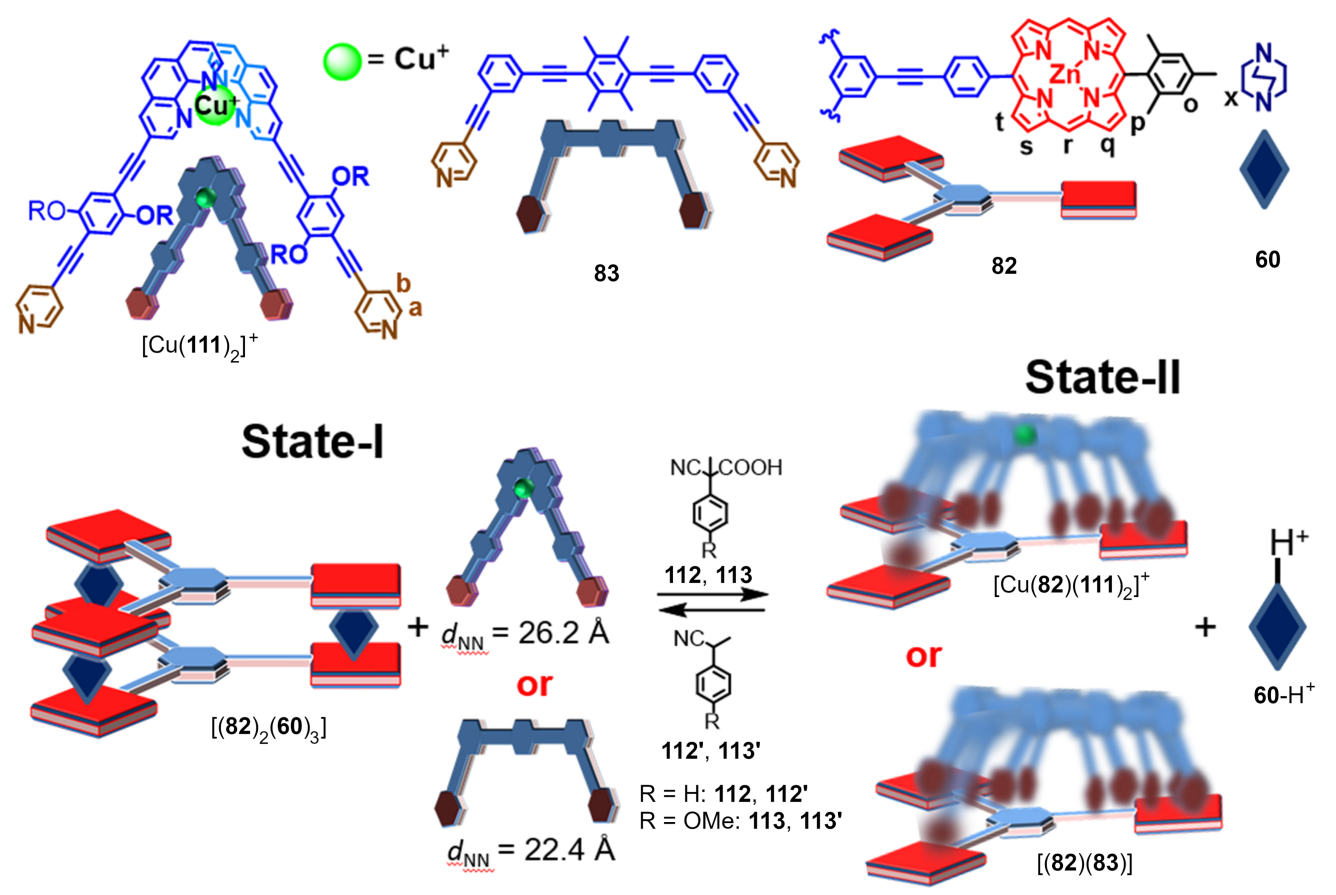
A chemically fueled catalytic system [112]. Figure was adapted from [112]. Copyright 2021 American Chemical Society. This content is not subject to CC BY 4.0.

**Figure 25:**
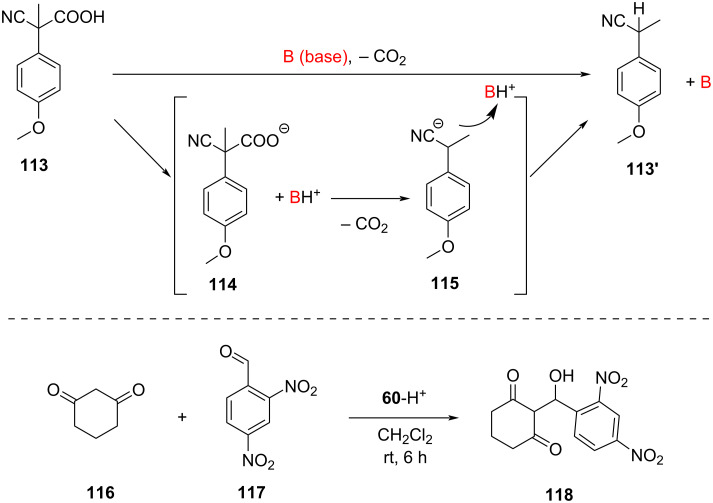
(Top) Operation of a fuel acid. (Bottom) Knoevenagel addition [112].

A completely different approach to switchable supramolecular catalysis made use of a supramolecular cage-to-device transformation under dissipative conditions (Figure 24) [112]. Notably, the dissipative conditions were realized by addition of a fuel acid [113–114] that surprisingly ignited a base-catalyzed Knoevenagel addition reaction (Figure 25).

As DABCO is a stronger binding ligand (log β = 7.20) [115] towards zinc porphyrin (ZnPor) than pyridine (log *K* = 4.45) [90] the reaction of ligand **111**, deck **82**, DABCO (**60**), and [Cu(CH_3_CN)_4_](PF_6_) (4:2:3:2) furnished the supramolecular cage [(**82**)_2_(**60**)_3_] with **60** = DABCO acting as pillars whereas biped [Cu(**111**)_2_]^+^ remained uncoordinated [112]. In an analogous self-assembly with **83** instead of [Cu(**111**)_2_]^+^, ligand **83** was left uncoordinated. When State-I was treated with TFA, then the reshuffling of the components afforded the slider-on-deck [Cu(**82**)(**111**)_2_]^+^ (or [(**82**)(**83**)]) and the monoprotonated DABCO (**60**-H^+^). Addition of DBU reversed the process. The bipeds in the slider-on-deck systems [Cu(**82**)(**111**)_2_]^+^ (*k*_298_ = 42.2 kHz) and [(**82**)(**83**)] (*k*_298_ = 32.2 kHz) move across the deck **82** and prevent binding of the protonated DABCO at the ZnPor binding sites. Use of the fuel acid **112** or **113** instead of applying the TFA/DBU acid/base combination leads initially to protonation of DABCO but due to the decarboxylation of **114** the resulting strong base **115** reclaims the proton back (Figure 25, top). As a result, State-II may be afforded under dissipative conditions.

**Figure 26:**
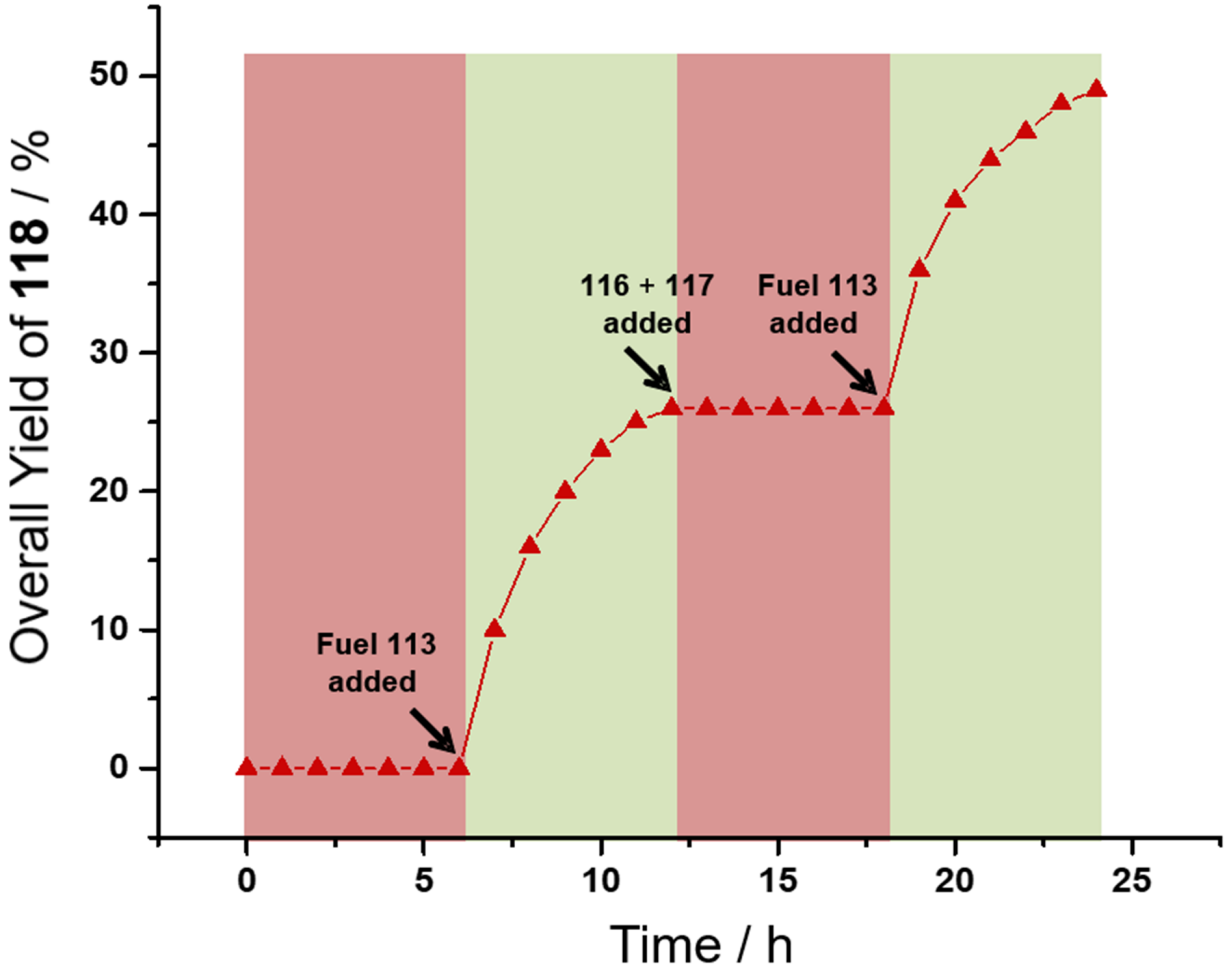
Development of the yield of Knoevenagel product **118** in a fueled system [112]. Figure was reprinted with permission from [112]. Copyright 2021 American Chemical Society. This content is not subject to CC BY 4.0.

A surprising facet of this State-I/State-II interconversion (Figure 24) [112] was the finding that the protonated DABCO (**60**-H^+^) was a rather efficient base catalyst for a Knoevenagel addition (Figure 25, bottom). Several control experiments excluded the possibility that the reaction was triggered by acid or by alternative pathways. The remaining unprotonated nitrogen in monoprotonated DABCO (**60**-H^+^) hence is a sufficiently strong base for the reaction of **116** and **117**. Finally, the State-I/State-II interconversion in the presence of **116** and **117** was triggered by addition of the fuel acid. The traces in Figure 26 show the amount of Knoevenagel addition product **118** within two subsequent pulses of the fuel.

In summary, the examples listed in this subchapter shed some light on the manifold opportunities for switchable ON/OFF catalysis controlled by supramolecular interconversions. Switchable catalysis decided by an ensemble of communicating molecules, which act as an information system and control the switching states, remodels somehow the situation in a living cell, where a desired event is only ignited when several signaling parameters agree. Explicitly, the subchapter demonstrates three distinct examples, in which metal–ligand coordination was shuffled by addition/removal of either metal ions or acid. To (re)shuffle metal–ligand coordination by acid/base generates a number of further options. In particular, the protocol of acid addition and removal (upon base addition) may be elegantly replaced by using a suitable fuel acid thus opening the field of supramolecular catalysis for out-of-equilibrium processing.

### Toggling between intra- and intermolecular complexation in nanoswitches

Heteroleptic complexation does not only open the way to multicomponent assembly but also to switchable catalysis in coordination-based toggles. An important and widely applicable protocol is the weak-link approach (WLA) that was developed and exploited by Mirkin [116]. It is based on the association/dissociation of hemilabile ligands bound to a metal center. Through the addition of secondary ligands, the weakly coordinated donor sites are substituted which allows an opening of the switch from a rigid-closed to a flexible semi-open form [117]. A common protocol used addition/removal of strongly binding monodentate ligands (CO or Cl^‒^) at rhodium centers to dissociate/reassociate the weakly coordinated donor, for instance in tweezer-type structures [118].

**Figure 27:**
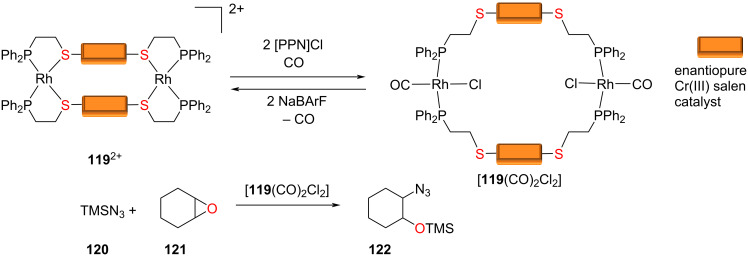
Weak-link strategy to increased catalytic activity in epoxide opening [119]. Figure was adapted from [24]. Copyright 2019 American Chemical Society. This content is not subject to CC BY 4.0.

An early contribution by Mirkin (Figure 27) described the opening of the closed structure **119**^2+^ to the open form [**119**(CO)_2_Cl_2_] that showed a two-fold activity in the catalytic opening of epoxide **121** to **122** [119].

**Figure 28:**
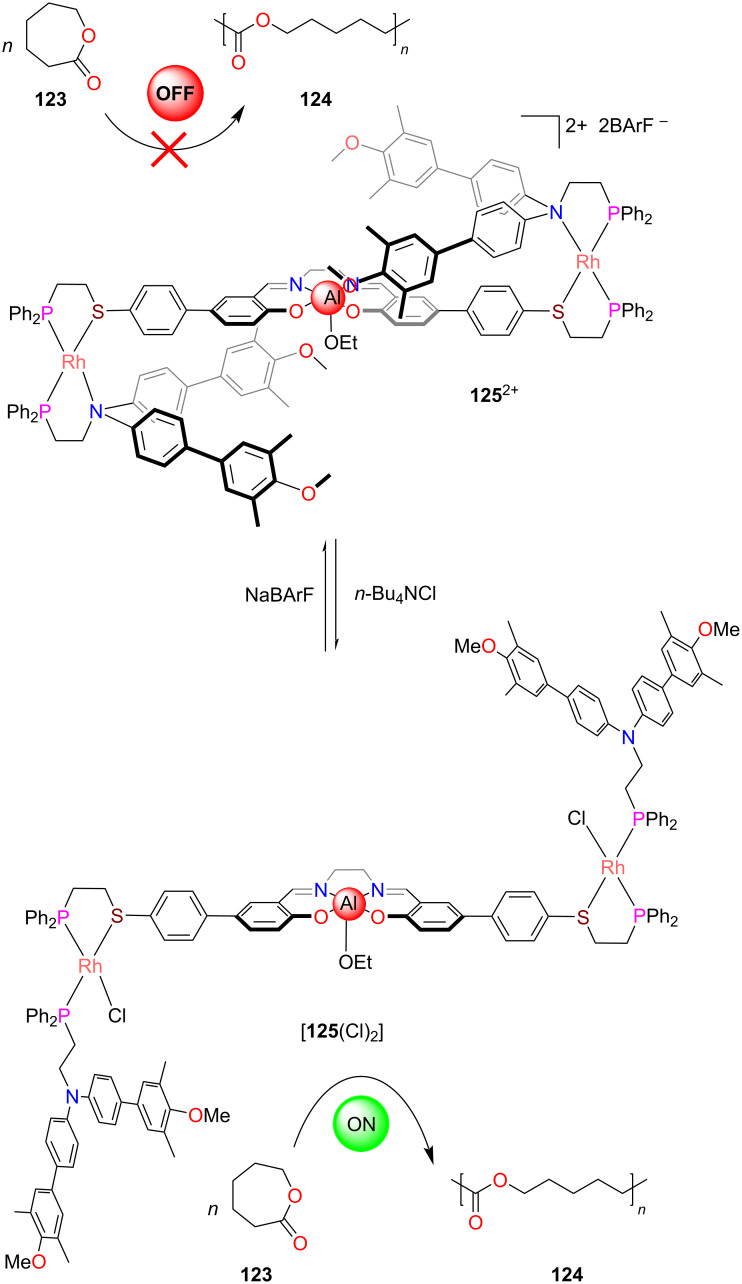
A ON/OFF polymerization switch based on the weak-link approach [118]. Figure was reprinted with permission from [24]. Copyright 2019 American Chemical Society. This content is not subject to CC BY 4.0.

In another example by Mirkin, a well-known aluminum(III) salen catalyst was hidden in switch **125**^2+^ [118] between sterically demanding biphenyl rings preventing ε-caprolactone from accessing the catalytic site (Figure 28). As a result, the polymerization of ε-caprolactone (**123**) was switched OFF. Addition of chloride anions from *n*-Bu_4_NCl led to the substitution of the tertiary amine ligand at both rhodium centers generating the semi-open form [**125**(Cl)_2_]. Since the aluminum(III) salen center became now exposed, the catalytic polymerization of ε-caprolactone (**123**) was turned ON. The chloride was readily removed by adding sodium tetrakis[(3,5-trifluoromethyl)phenyl]borate (NaBArF) and as result catalysis was turned OFF again.

Along this protocol, Mirkin et al. have developed a variety of allosterically modulated catalysts that allow ON/OFF reaction control in photoredox catalysis [120], phosphate diester transesterification [121], Friedel–Crafts reaction, ring opening of epoxides, oligomerization [116], and acyl-transfer reactions [122–123].

While there are further examples by Mirkin [124–126], Schmittel [127–128], and others [129–130] that operate at the borderline of dynamic heteroleptic complexation events, the supramolecular cases selected in the following are characterized by the reshuffling of inter- and intramolecular coordination events to realize different toggling states for catalysis [131].

**Figure 29:**
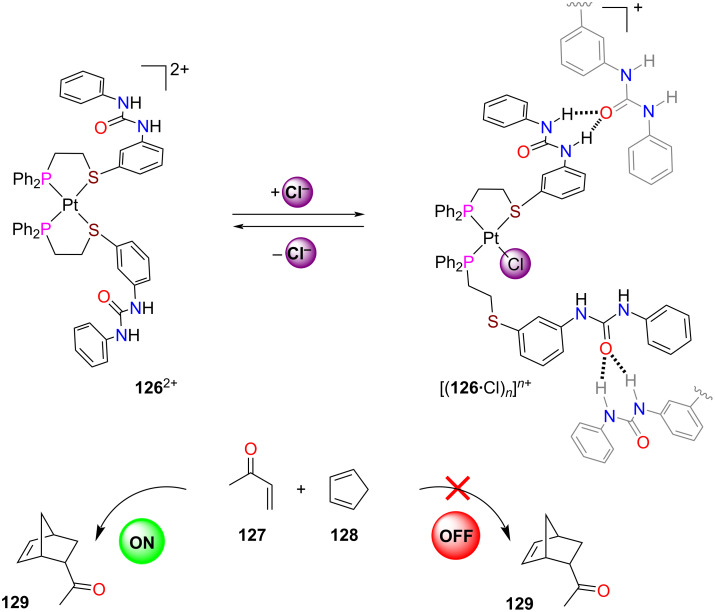
A weak-link switch turning ON/OFF a Diels–Alder reaction [132]. Figure was reprinted with permission from [24]. Copyright 2019 American Chemical Society. This content is not subject to CC BY 4.0.

Using addition and removal of chloride, the Mirkin group reversibly and quantitatively toggled the platinum(II)-based switch **126**^2+^ between a homo- and heteroligated form (Figure 29) [132]. In the closed platinum(II) complex **126**^2+^, the urea units were available for activation of butenone (**127**) by hydrogen bonding. As a result, the Diels–Alder reaction of cyclopentadiene (**128**) and **127** was catalyzed. Upon addition of *n*-Bu_4_NCl, the open form was afforded that aggregated to oligomers [(**126**•Cl)*_n_*]*^n^*^+^ through intermolecular hydrogen bonding at the urea moieties. Now, activation of **127** stopped and catalysis was turned OFF. Catalysis was turned back ON after trapping of the chloride ions with NaBArF.

The next example bridges in some way the gap between the preceding (Figure 23) and the present chapter because it addresses again catalysis with two catalytically active copper(I) centers held at a defined distance, now though for proximity catalysis using acyl transfer. The utility of this approach was demonstrated when a duo of catalysts was used for achieving substrate selectivity [133].

**Figure 30:**
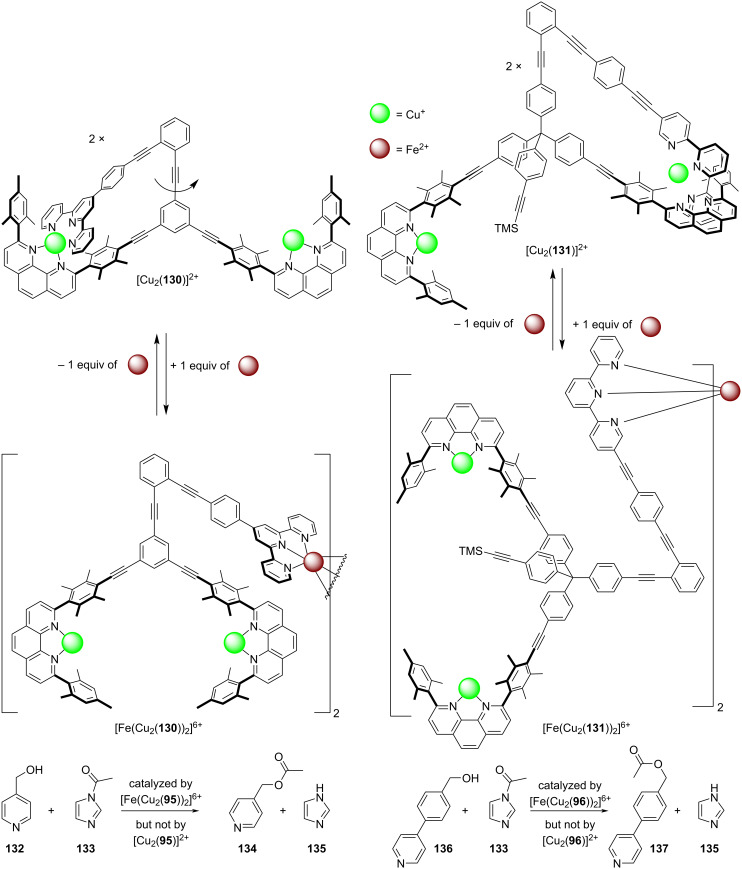
A catalyst duo allowing selective activation of one of two catalytic acylation reactions [133] upon substoichiometric amounts of iron(II). For explanations, see text. Redrawn from reference [133].

In detail, nanoswitch [Cu(**130**)]^+^ was transformed into the slow rotor [Cu_2_(**130**)]^2+^ (*k*_298_ = 1.34 s^−1^) upon the addition of a second equiv of copper(I) ions (Figure 30). When 0.5 equiv of iron(II) was added, the rotator arm got involved in iron(II) bis(terpyridine) complexation affording [Fe(Cu_2_(**130**))_2_]^6+^. An analogous transformation was seen for [Cu(**131**)]^+^ → [Cu_2_(**131**)]^2+^ → [Fe(Cu_2_(**131**))_2_]^6+^.

When hydroxymethylpyridine **132** and the acetylation agent **133** (Figure 30) were reacted in the presence of the copper(I)-loaded rotor [Cu_2_(**130**)]^2+^ no reaction was detected. In contrast, the dimeric complex [Fe(Cu_2_(**130**))_2_]^6+^, formed after addition of iron(II) ions, offers two cavities each with two copper(I) ions to pre-bind both **132** and **133** at an optimal reaction distance. It was no surprise, that due to the increased local concentration, the acetylation **132** + **133** → **134** did rapidly take place. Similarly, the bigger dimeric nanoswitch [Fe(Cu_2_(**131**))_2_]^6+^ catalyzed the acetylation of the larger substrate **136** due to size-matching [133]. In order to test the reversibility, both switches were toggled by adding and removing the iron(II) ions over 2.5 cycles demonstrating that ON/OFF catalysis was reversible and reproducible.

Interestingly, the nanoswitches [Cu_2_(**130**)]^2+^ and [Cu_2_(**131**)]^2+^ (1:1) could be selectively addressed in the presence of all substrates and reagents (**132**, **133**, and **136**). Upon addition of substoichiometric amounts of iron(II), predominantly nanoswitch [Cu_2_(**131**)]^2+^ reacted to afford [Fe(Cu_2_(**131**))_2_]^6+^ which selectively turned on the reaction affording **137**. As a result, this example highlights a sophisticated case of a multicatalyst system that can select between substrates of essentially identical reactivity but different size.

**Figure 31:**
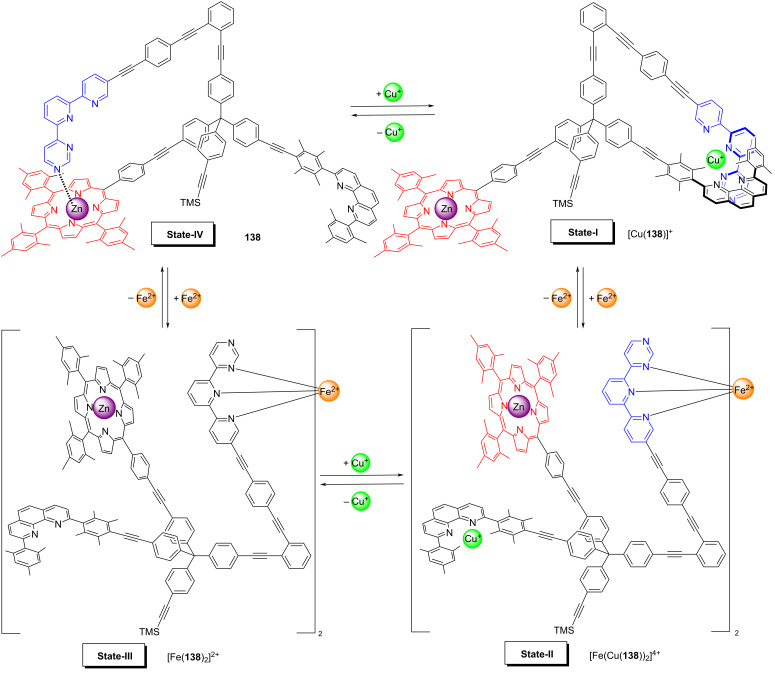
A four-state switchable nanoswitch (redrawn from [134]).

A spectacular and unique example of toggling catalysis was demonstrated in nanoswitch **138** (Figure 31) with its four distinct switching states [134]. In State-I, i.e. [Cu(**138**)]^+^, an intramolecular HETTAP complex between a copper(I) phenanthroline and a terpyridine site was realized. Upon addition of 0.5 equiv of iron(II) ions the HETTAP interaction opened up and the “dimeric” bishomoleptic nanoswitch [Fe(Cu(**138**))_2_]^4+^ was furnished in State-II held together by a bis(tpy) iron(II) complexation. Due to the eradication of the HETTAP binding, the copper(I) ions were left coordinatively frustrated, which made them potentially available for catalysis of a click reaction. In the next switching step, the removal of copper(I) ion led to State-III, i.e. [Fe(**138**)_2_]^2+^. The ensuing removal of iron(II) ions broke down the bis(tpy) complex [Fe(**138**)_2_]^2+^ and afforded State-IV. The latter state was characterized by a closed structure of the nanoswitch **138** due to the intramolecular *N*_pym_ → ZnPor binding (pym = pyrimidine). The full switching cycle using addition/removal of ions was repeated without fatigue.

**Figure 32:**
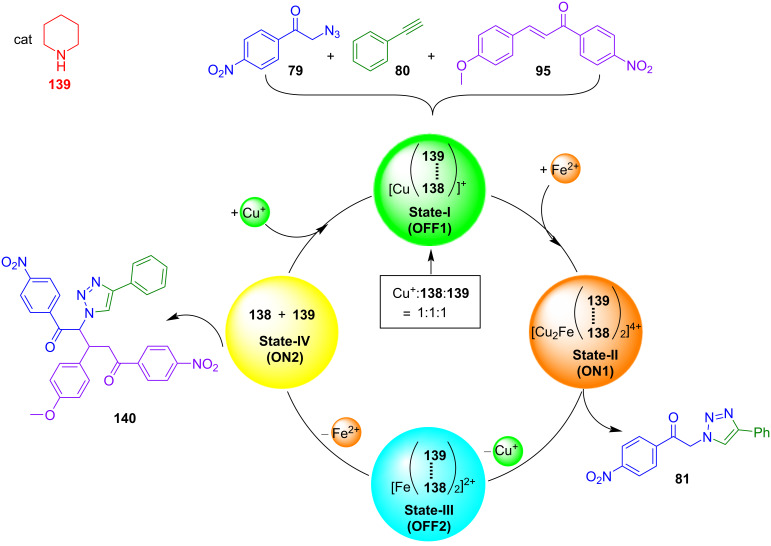
Sequential catalysis as regulated by nanoswitch **138** and catalyst **139** in the presence of metal ions (redrawn from [134]).

The above sequence of four switching states has been demonstrated to activate sequential catalysis (Figure 32), once the protocol is executed in the presence of piperidine (**139**) as a catalyst and the reactants **79**, **80**, and **95** [134]. Due to the “free” ZnPor unit in State I, catalyst **139** was strongly bound at the ZnPor unit of [Cu(**138**)]^+^, while the copper(I) ion was firmly encapsulated in a HETTAP binding site. Thus, State-I should be catalytically inactive. Upon addition of iron(II), the tpy unit of the HETTAP unit became involved in the bishomoleptic iron(II) terpyridine complex [Fe(Cu(**138**))_2_]^4+^, while the copper(I) ions were exposed for catalysis in State-II. Indeed, in this state 50% of the click product **81** was formed. Removal of copper(I) and formation of State-III stopped the click catalysis. The ensuing removal of iron(II) ions finally afforded nanoswitch **138** (= State-IV). Due to the intramolecular linkage of the azaterpyridine to the ZnPor unit, the piperidine that was firmly bound in States I → III, was now released from the ZnPor unit. Remarkably, product **81** formed in State-II now underwent a catalyzed Michael addition to provide **140** in 28% yield. When the catalytic cycle was repeated, it fully reproduced the yields of the first cycle, thus demonstrating that a catalytic eleven-component machinery may work without destructive interference despite the large number of functional groups in the switches, reagents, and products [134].

**Figure 33:**
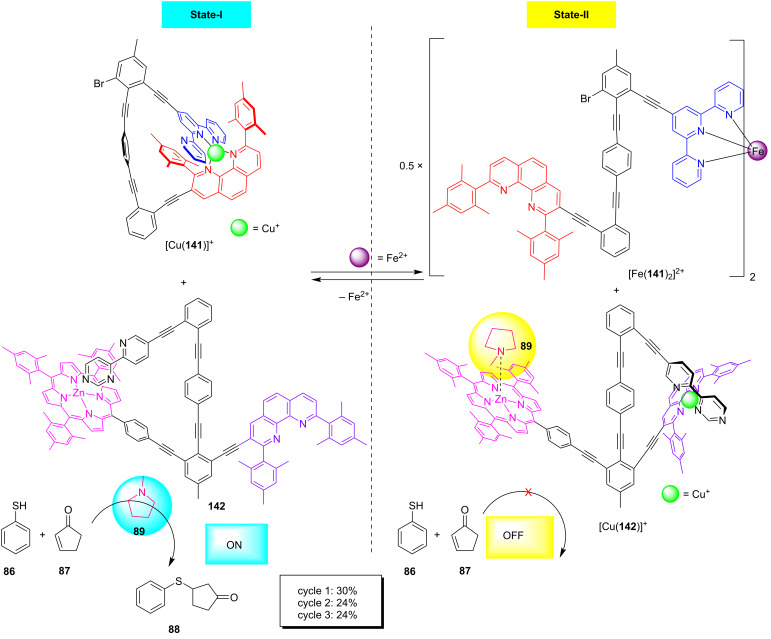
Remote control of ON/OFF catalysis administrated by two nanoswitches through ion signaling (redrawn from [135]).

The following example (Figure 33) does not reach the complexity of the above nanoswitch system, but it is remarkable as it involves a remote control of catalysis [135]. Here, the complex [Cu(**141**)]^+^ with its heteroleptic HETTAP binding site controlled the switching state of nanoswitch **142** by using fully reversible communication via ion signaling. State-I was characterized by a clean self-sorting of the copper(I) ions resulting in [Cu(**141**)]^+^ + switch **142**. In the presence of *N*-methylpyrrolidine (**89**), a conjugate addition was observed in State-I. However, upon addition of iron(II) (0.5 equiv) ligand **141** became involved in the bishomoleptic iron(II) terpyridine complex [Fe(**141**)_2_]^2+^ with the effect that now weakly bound copper(I) ions travelled to nanoswitch **142**. In the thus formed complex [Cu(**142**)]^+^ the azabipyridine dissociated from the ZnPor binding site to generate a HETPHEN complexation site for copper(I). With the ZnPor unit being freed from intramolecular binding, it now served as coordination site for the *N*-methylpyrrolidine (**89**), preventing the latter from catalyzing the conjugate addition [135]. As a result, catalysis was stopped in State-II. The cycle could be repeated two more times, however, generating a lower yield (Figure 33). The reduction of the yield in cycles two and three was traced to the slow switching of State-II to State-I.

In summary, this subchapter highlighted switching systems that were built using dynamic heteroleptic complexation together with supramolecular dissociation/association events for regulating catalysis. It is noteworthy that switching in all cases went along with a major nanomechanical reorganization of one (or all) constituents.

## Conclusion

Supramolecular catalysis is an emerging field that has attracted a lot of attention recently. Its comprehensive coverage encompasses several fields [3], however, still with a main focus on catalysis that is promoted by discrete cages, capsules, and in a variety of confined environments. The present short account with a focus on dynamic heteroleptic metal complexation seeks to demonstrate that (metallo)supramolecular catalysis has many more opportunities to offer. While the examples in the first subchapter illustrate the much higher diversity of heteroleptic over homoleptic cages, the following subchapters above all exemplify that supramolecular chemistry extends beyond the single "supramolecular structure", as complex it may be, thus reaching far into the field and concepts of systems chemistry [30,136] and information science [137–138]. For instance, toggling between and dynamic exchange within supramolecular structures add features to catalysis as ON/OFF or UP/DOWN regulation of catalytic activity. If these factors will be consequently developed, they should eventually open opportunities to autonomous catalytic systems.

A look at systems biology, where enzymatic catalysis is tightly regulated by various chemical messengers to enable life, can serve as a guide. In this regard, we expect that upcoming endeavors will increasingly focus on the development of artificial systems that can make autonomous decisions and that energetically operate out-of-equilibrium.
